# Addressing the Intracellular Vestibule of the Plasmodial Lactate Transporter PfFNT by p-Substituted Inhibitors Amplifies In Vitro Activity

**DOI:** 10.1021/acs.jmedchem.4c01674

**Published:** 2024-10-03

**Authors:** Cornelius Nerlich, Finn Tiedjens, Robin Hertel, Björn Henke, Susan Häuer, Lea S. Panitzsch, Kerrin Hansen, Ole Franck, Antonio Mete, Didier Leroy, Dennis Schade, Christian Peifer, Stefan Hannus, Frank Becker, Sergio Wittlin, Tobias Spielmann, Eric Beitz

**Affiliations:** † Department of Pharmaceutical and Medicinal Chemistry, Christian-Albrechts-University of Kiel, Gutenbergstr. 76, 24118 Kiel, Germany; ‡ Bernhard-Nocht-Institute for Tropical Medicine, Bernhard-Nocht-Str. 74, 20359 Hamburg, Germany; § Intana Bioscience GmbH, Lochhamer Str. 29a, 82152 Planegg, Germany; ∥ Medsyndesign Ltd, ATIC, 5 Oakwood Drive, LE11 3QF Loughborough, U.K.; ⊥ R&D Department/Drug Discovery, ICC, Medicines for Malaria Venture (MMV), 20 Route de Pré Bois, 1215 Geneva 15, Switzerland; # Swiss Tropical and Public Health Institute, Kreuzstr. 2, 4123 Allschwil, Switzerland; ¶ University of Basel, 4003 Basel, Switzerland

## Abstract

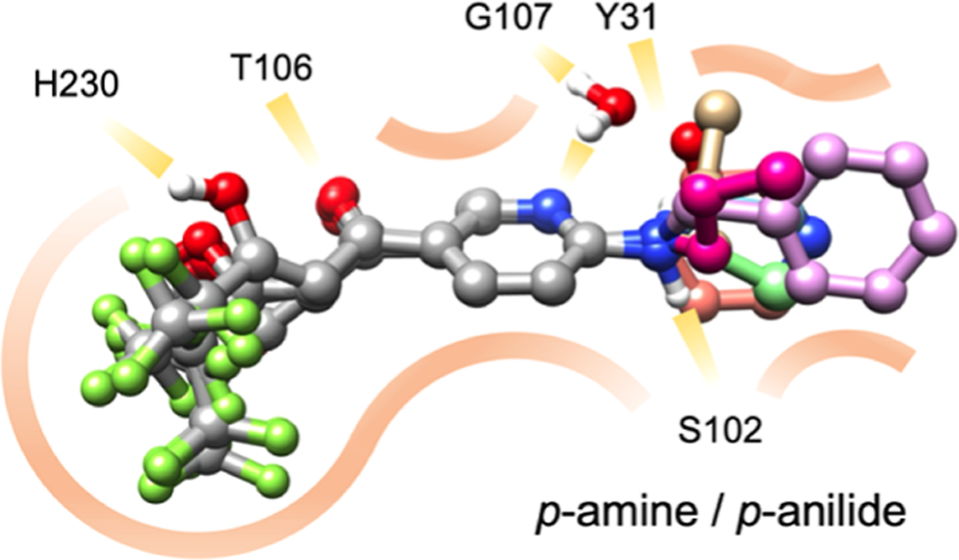

Inhibition of the lactate transporter PfFNT is a valid novel mode of action against malaria parasites. Current pyridine-substituted pentafluoro-3-hydroxy-pent-2-en-1-ones act as substrate analogs with submicromolar EC_50_ in vitro, and >99.7% activity in mice. The recently solved structure of a PfFNT-inhibitor complex visualized the binding mode. Here, we extended the inhibitor layout by series of amine- and anilide-linked pyridine p-substituents to generate additional interactions in the cytoplasmic vestibule. Virtual docking indicated hydrogen bonding to Tyr31 and Ser102. Fluorescence cross-correlation spectroscopy yielded respectively enhanced target affinity. Strikingly, the in vitro activity increased by 1 order of magnitude to 14.8 nM at negligible cytotoxicity. While *p*-amine substitutions were rapidly metabolized, the more stable *p*-acetanilide cleared 99.7% of parasites at 4 × 50 mg kg^–1^ in a mouse malaria model. Future stabilization of the p-substitution against metabolism may translate the gain in in vitro potency to the in vivo situation.

## Introduction

Drugs to fight malaria have proved particularly successful when they modify general physicochemical properties of the parasite’s compartmental fluids or cytosol, even though their exact mechanism of action is not fully understood. In this sense, chloroquine shifts the pH of the food vacuole toward alkaline and blocks heme detoxification.^1^ Artemisinin derivatives elevate the intracellular oxidative potential via a common peroxo moiety possibly resulting in lethal cell stress.^2^ Cipargamin raises the cytosolic osmolarity by blocking the release of sodium cations via the Na^+^/H^+^-ATPase, ATP4, leading to swelling and cell death.^3^ Inhibition of ATP4 concomitantly disturbs the cytosolic pH homeostasis supposedly affecting multiple vital enzymatic and transport processes. We and others discovered^4,5^ and developed^6,7^ a class of potent inhibitors of the plasmodial lactate/H^+^ symporter, PfFNT,^8^ that acidify the cytosol^9^ and interfere with the energy metabolism of the parasites in the asexual blood stage.^10^ The compounds kill the parasites presumably by eliciting multitarget effects due to a drop in the cytosolic pH and loss of ATP as a fuel.

**Figure 1:**
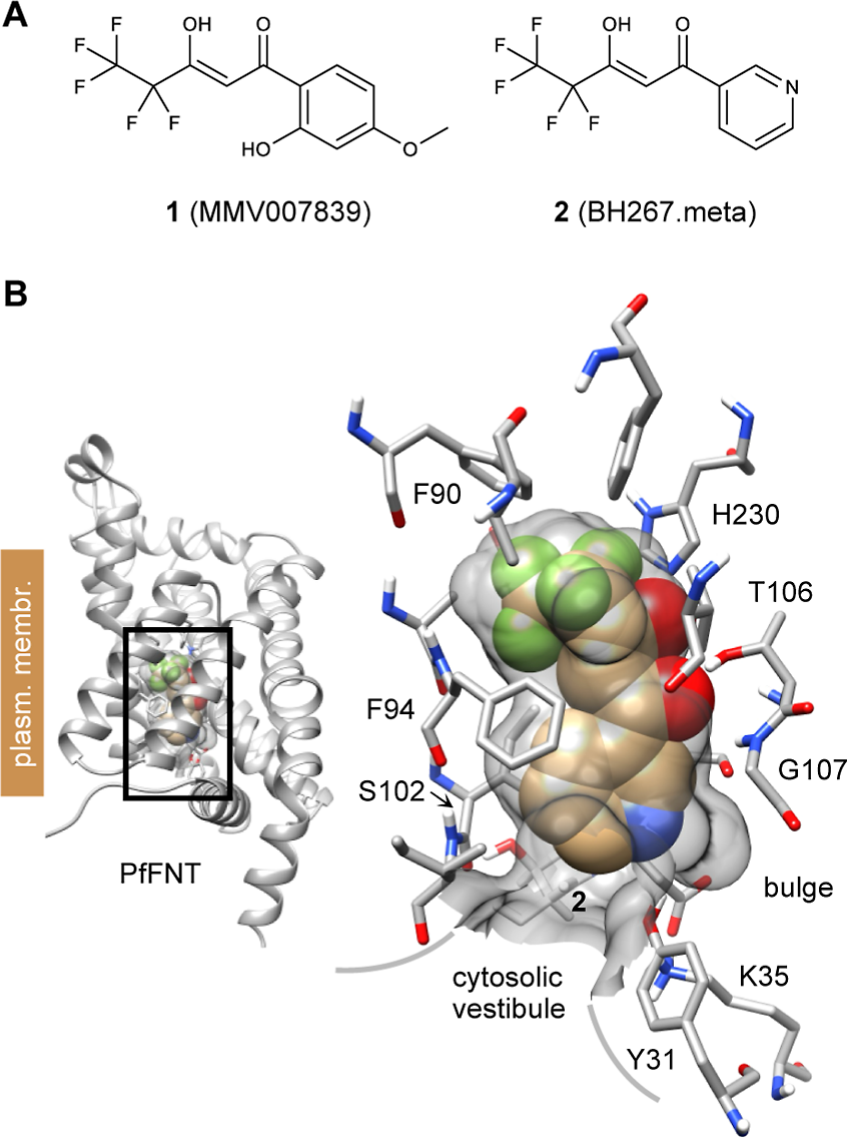
Current inhibitors of the *Plasmodium falciparum* (*P. falciparum*) lactate/H^+^ transporter, PfFNT, and binding mode. (A) Structures of the malaria box compound MMV007839 **1**, and the G107S resistance-circumventing BH267.meta **2**. (B) Binding of **2** to PfFNT (PDB# 7e27). Prominent amino acid side chains are labeled. His230 and Thr106 form hydrogen bonds with the vinylogous acid moiety of **2**; the remaining residues define a largely lipophilic surface (gray). The bulge next to Gly107 is water molecule-sized.

The most potent known PfFNT inhibitors share a 3-hydroxy-prop-2-en-1-one vinylogous acid moiety flanked by a short lipophilic fluoroalkyl chain and an aromatic ring^10^ (Figure 1A) achieving in vitro activities in the EC_50_ range of 150–250 nM. The compounds act as substrate analogs by mimicking two consecutive states of the transport mechanism in one linear molecule: the anionic lactate state for attraction (vinylogous acid part), and the protonated lactic acid state (fluoroalkyl chain) for entering the lipophilic center of the transporter.^4^ The binding mode was deduced from enforced in vitro selection of a PfFNT G107S resistance mutation in conjunction with structure activity relationships,^6^ and was confirmed by elucidating the protein structure^11^ in the presence of the inhibitor MMV007839 **1** (discovered from the malaria box compound collection),^12^ and by modeling of BH267.meta **2** into the active site of the PfFNT protomer (Figure 1B).

The PfFNT inhibitor binding site is largely lipophilic. Only His230, which is an almost invariable signature residue of the strictly microbial FNT-type monocarboxylate transporters,^13^ and the strongly conserved Thr106 form hydrogen bonds with the inhibitor. It is of note that, in contrast to other transporter proteins, the channel-like structure of PfFNT is highly rigid and does not undergo major conformational changes.^11^ Access from the cytosol to the transporter core is enabled by a single 90° swing-out movement of the Phe94 benzyl side chain (Figure 1B). Likewise, Phe90 is supposed to control access from the extracellular side.^14^

The aim of this study was to make use of the new structure information to find PfFNT inhibitors with enhanced target affinity and an in vitro activity below 100 nM.

## Results

### Current Inhibitors Effectively Fill the PfFNT Binding Pocket above Tyr31

The PfFNT inhibitor BH267.meta **2** in its bound state occupies 173.6 Å^3^ of the available volume of approximately 190 Å^3^ in the binding pocket above Tyr31 (Figure 1B). To evaluate structural variability within the binding pocket, we modified the fluoroalkyl moiety, the central vinylogous acid part, and the type of the aromatic ring. We determined (i) the target binding affinity by fluorescence cross-correlation spectroscopy (FCCS), (ii) the inhibition of PfFNT transport functionality for ^14^C-lactate in yeast, and (iii) the in vitro activity using *Plasmodium falciparum* 3D7 parasite cultures in erythrocytes. Data are shown in Table 1 in comparison to the reference compounds **1** and **2** (see Figures S1A and S2A for *K*_*i*_/IC_50_ curves).

**Table 1:**
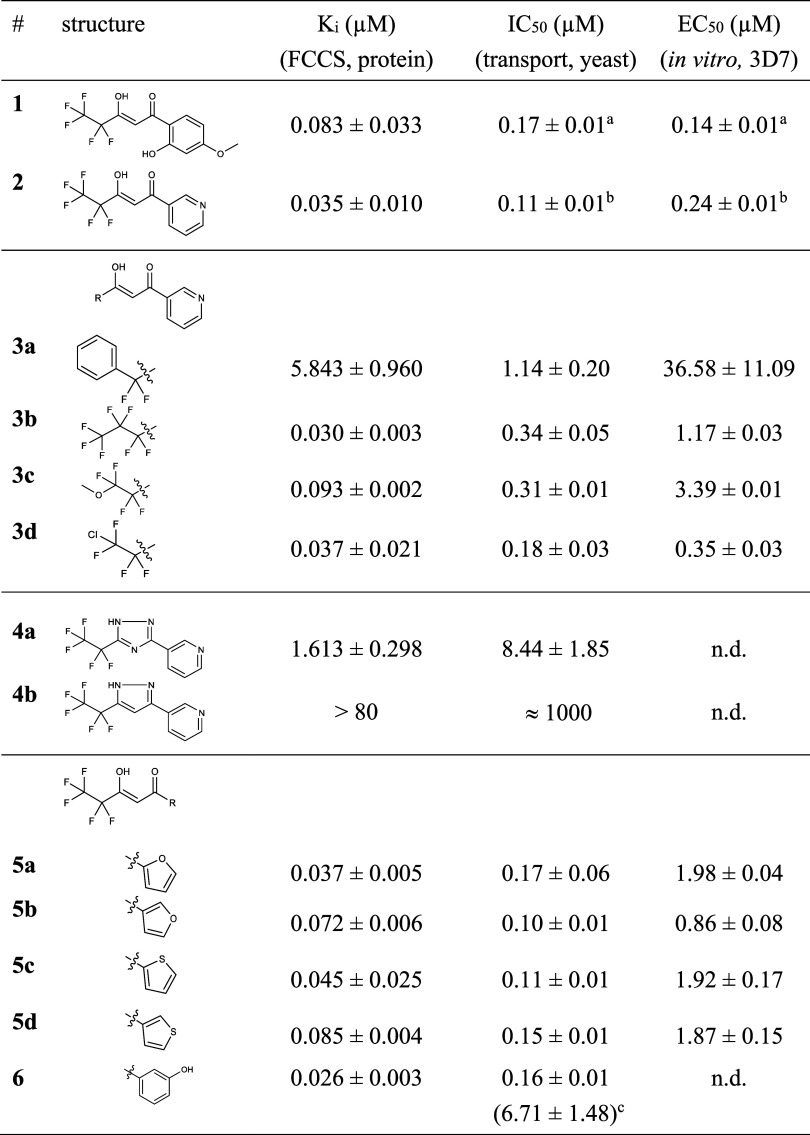
Affinity and Activity of PfFNT Inhibitors Restricted to the Inner Binding Pocket

a Taken from Golldack^4^ et al. 2017.

b From Walloch^6^ et al. 2020.

c PfFNT G107S.

Replacement of the trifluoromethyl moiety by a phenyl ring (**3a**) enlarged the molecular volume to 208.0 Å^3^ and strongly decreased both affinity and activity (Table 1). Three more modifications yielding a smaller increase in volume, i.e. introduction of one additional difluoromethylene unit (**3b**, 194.3 Å^3^), and substitution of one terminal fluorine atom by methoxy (**3c**, 193.8 Å^3^) or chlorine (**3d**, 191.4 Å^3^) were tolerated by the binding pocket as seen by high but not increased binding affinity over **1** and **2**. However, only **3d** gave submicromolar in vitro activity in erythrocytes. The latter is influenced by additional, target-independent pharmacokinetic parameters, for example uptake of the inhibitor into the parasites and free compound concentration vs plasma protein binding, which need to be taken into consideration for future development.

Bioisosteric replacement of the vinylogous acid part (p*K*_a_ ≈ 5) by a triazole (**4a**) or a diazole moiety (**4b**) was expected to maintain the hydrogen bonding pattern, while lowering the acid strength to p*K*_a_ ≈ 8 and 10, respectively. In line with the protonation-dependent two-stage transport mechanism of PfFNT,^14^ the mainly neutral compounds **4a** and **4b** exhibited low or negligible affinity and activity (Table 1).

Changing the aromatic system to furan or thiophene (**5a**–**d**) maintained the binding affinity and inhibition of yeast-expressed PfFNT in a similar range to BH267.meta **2**. The in vitro activity against 3D7 parasites, however, dropped to micromolar EC_50_ concentrations (Table 1). Eventually, we considered if the bulge in the surface of the binding pocket next to Gly107 (Figure 1B) may hold a water molecule. It is considered an entropic disadvantage, resulting in lower affinity, if a binding ligand isolates and entraps a water molecule in the pocket.^15^ Therefore, we prepared **6**, containing a 3-hydroxy-substituted benzene ring, to displace the putative water molecule. The affinity of 26 nM obtained, was indeed higher than that of the heterocyclic compounds (**2**, **3a**–**d**, **5a**–**d**) suggesting that these compounds coordinate via an entrapped water molecule to the backbone of Gly107. The rather small decrease in *K*_*i*_ obtained, however, suggests that there is only a moderate entropy penalty by isolating the water molecule. We forwent determination of the EC_50_ in vitro activity of **6**, since a 3-hydroxy substitution collides with the putative PfFNT Gly107Ser resistance mutation (IC_50_ 6.71 μM; Table 1, Figure S2A) that fills the exact site of the coordinated water^6^ preventing further development of a respective line of compounds.

### p-Substitution via Secondary Amines or Anilides Enables Low Nanomolar In Vitro Activity

**Figure 2:**
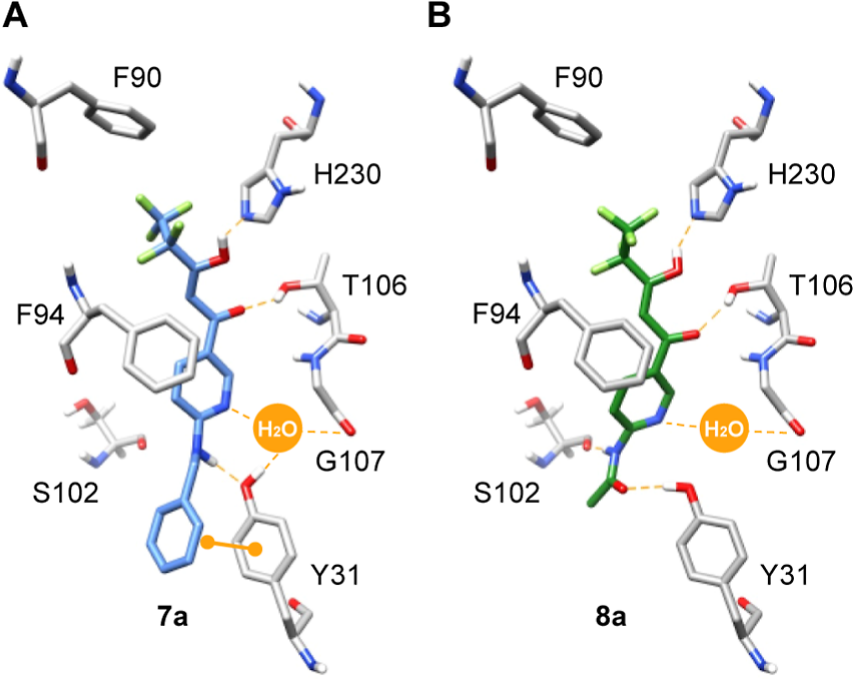
Virtual docking of BH267.meta (**2**) variants with p-substitutions. (A) Secondary amines (**7a**, benzylamine) appear to donate a hydrogen bond to Tyr31, and the benzylamine ring is within π-stacking distance. (B) Anilides (**8a**, acetanilide) may instead donate a hydrogen bond to the backbone of Ser102, and accept one from Tyr31.

With the inner binding pocket effectively filled by BH267.meta **2**, we extended the compounds in the p-position of the pyridine ring to interact with amino acid residues of the cytosolic vestibule of PfFNT. Here, the polar side chains of Tyr31, Lys35, and Ser102 appeared attractive (Figure 1B), because the vestibule widens considerably below Tyr31 toward the cytosol becoming more solvent exposed and less amenable to efficient ligand binding (Figure 1B). Virtual docking of **7a** carrying a *p*-benzylamine moiety and the acetanilide **8a** suggested additional hydrogen bond interactions to Tyr31 and the backbone of Ser102, respectively (Figure 2A,B). The benzylamine ring of **7a** may also, potentially, interact with the aromatic system of Tyr31 by π-stacking.

**Scheme 1:**
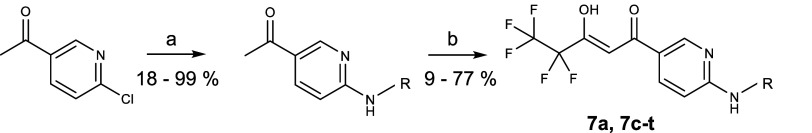
Synthesis of *p*-Amino-Variants of BH267.meta Reagents and conditions: (a) alkylamine e.g. benzylamine for **7a**, *i*-PrOH, 120 °C, 1.5 h; (b) ethyl pentafluoropropionate, NaOMe (30%), THF, 0–20 °C, o. n.

**Scheme 2:**
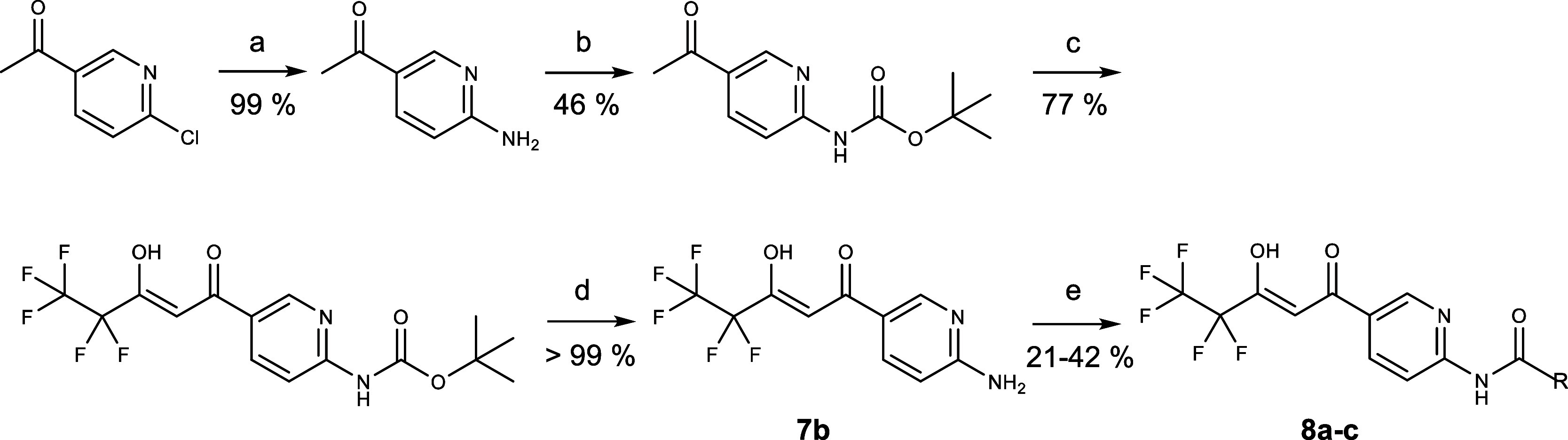
Synthesis of *p*-Anilide-Variants of BH267.meta Reagents and conditions: (a) NH_4_OH (33%), 160 °C, 5 h; (b) Boc_2_O, DMAP, TEA, DCM, r. t.; (c) ethyl pentafluoropropionate, NaOMe (30%), THF, 0–20 °C, o. n.; (d) TFA, DCM, 0 °C; (e) carboxylic acid anhydride e.g. acetic anhydride for **8a**, pyridine, 60–80 °C, 9–13 h.

**Figure 3:**
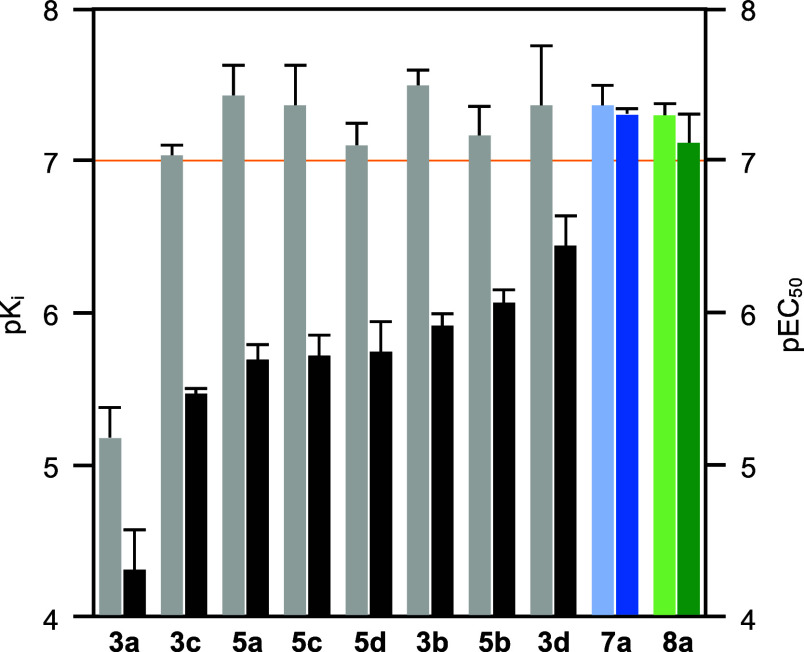
Target affinity vs in vitro activity of PfFNT inhibitors. Bars are colored light for PfFNT affinity (p*K*_i_) and dark for in vitro activity (pEC_50_). The orange line marks the 100 nM threshold. The secondary amine **7a** compound is shaded blue, the anilide **8a** green.

Synthesis of **7a** and **8a** (Schemes 1 and 2) and activity assays confirmed the good potential of the secondary amine and anilide PfFNT inhibitor compounds. Both exhibited high binding affinity with *K*_*i*_ values of 32 and 35 nM, respectively (Figure 3, Tables 2 and 3). The EC_50_’s of 77.9 and 49.8 nM, in the in vitro activity on 3D7 parasites were well below the targeted 100 nM threshold (pEC_50_ > 7). **7a** and **8a** exhibit a smaller difference between target affinity and in vitro activity compared to earlier compounds (Figure 3). Apparently, the secondary amine and anilide moieties introduced compound properties that not only contribute to target affinity but are favorable for high in vitro activity.

**Table 2:**
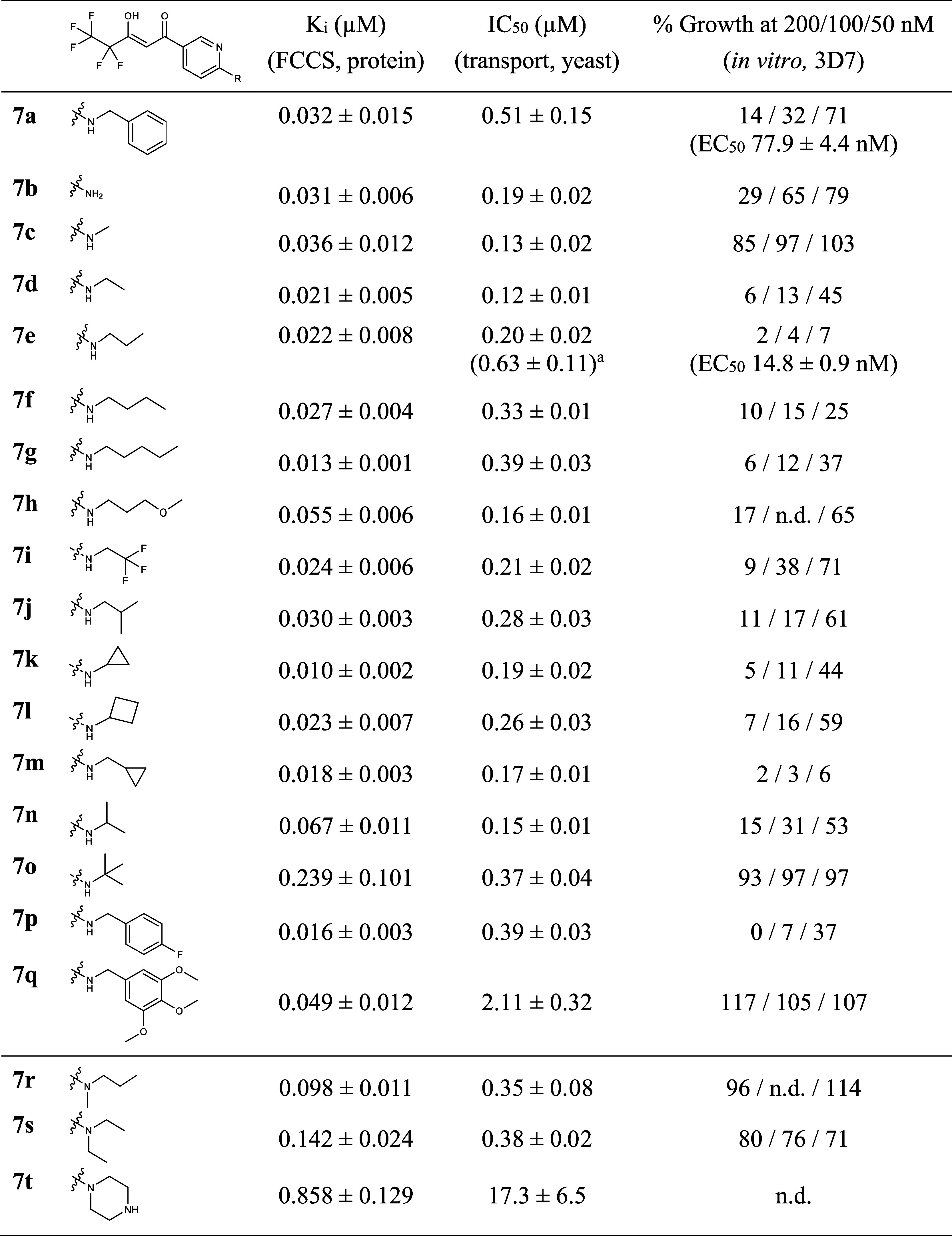
Affinity and Activity of the *p*-Amino-Substituted Line of PfFNT Inhibitors

a PfFNT G107S.

**Table 3:**
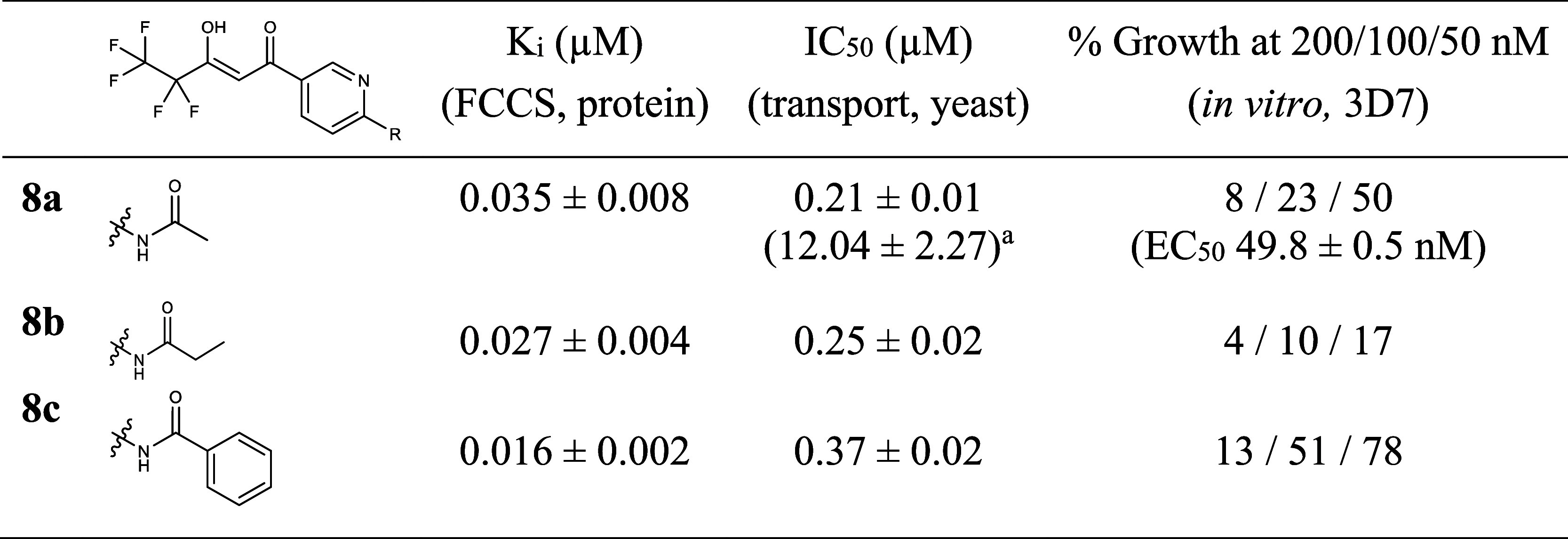
Affinity and Activity of *p*-Anilide-Substituted PfFNT Inhibitors

a PfFNT G107S.

### Line Extension of *p*-Amine- and *p*-Anilide-Substituted PfFNT Inhibitors

The synthetically readily accessible **7a**, allowed us to generate a homologous series of secondary and tertiary amines (**7c**–**t**, Scheme 1) as well as the unsubstituted aniline (**7b**, Scheme 2). This allowed us to explore the target affinity and inhibitory activity, in terms of PfFNT lactate transport and parasite growth, for a wide range of modifications (Table 2, Figures S1B and S2B).

With the exception of **7o** carrying a quaternary carbon, the compounds from the secondary amine series (**7c**–**q**) showed excellent *K*_*i*_ values in the range of 10–67 nM. In line with these data, PfFNT ^14^C-lactate transport in the yeast system was also potently inhibited with IC_50_ values from 120 to 390 nM. Here, only the large trimethoxy-substituted benzamine variant **7q** was less efficient at 2.11 μM, possibly due to hampered uptake into the cells. The tertiary amine variants **7r** and **7s** exhibited lower affinity of 98 and 142 nM, respectively, supporting the predicted hydrogen bond donor function of the amine to the Tyr31 hydroxyl (Figure 2A). It cannot be excluded that the branching at the amine nitrogen may generate additional steric disadvantages because the ethyl branch of **7s** increased the effect over the smaller methyl of **7r**. Introduction of a basic amine in **7t** was strongly detrimental to PfFNT affinity.

**Figure 4:**
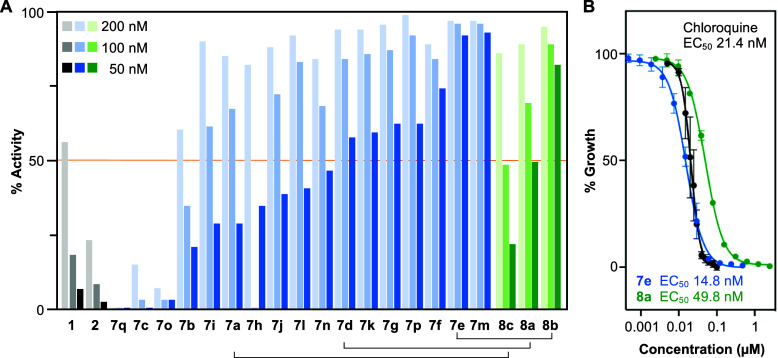
In vitro activity of *p*-amino- and *p*-anilide-substituted variants of BH267.meta. (A) The activity on 3D7 cultures of the amine is shown (blue) and anilide series (green) of PfFNT inhibitors compared to **1** and **2** at 200, 100, and 50 nM. The brackets link structurally analogous compounds. (B) EC_50_ determination of **7e**, **8a**, and chloroquine.

To gain data on the in vitro activity of the p-substituted compound series, we used a screening-type setup in which growth of cultured 3D7 parasites was determined at three single compound concentrations of 200, 100, and 50 nM. Here, the reference compounds MMV007839 **1** and BH267.meta **2** yielded growth levels >90%, i.e. <10% inhibitory activity, at 50 nM (Figure 4A). Of the secondary amines, **7e** (*n*-propylamine) and **7m** (cyclopropylmethylamine) stood out by resulting in only very low parasite growth of 7% and 6%, respectively, i.e. 93% and 94% inhibitory activity, at 50 nM compound concentration (Figure 4A, Table 2). Generally, linear alkyl moieties of three or more carbons and flat structures (tertiary carbons or phenyl) gave highly active compounds with estimated EC_50_ values around or below 50 nM as deduced from % activity at 50 nM compound concentration (Figure 4A, orange line). Determination of the true EC_50_ of **7e** and the acetanilide **8a** yielded 14.8 and 49.8 nM, respectively; in comparison chloroquine, when assayed in the same system, gave an EC_50_ of 21.4 nM (Figure 4B). We followed up these results by preparing the propionanilide **8b** and benzanilide **8c**, and these showed comparable affinity and activity as their structural analogs **7e** and **7a** (Figure 4A, Table 3, Figures S1C and S2C). Assaying representative compounds against the PfFNT G107S resistance mutant revealed differentiated activities. The secondary amine **7e** was marginally affected while the anilide **8a** was strongly affected (Tables 2 and 3, Figure S2B,C).

Together, introduction of a *p*-amino or *p*-anilide moiety increased the in vitro activity by 1 order of magnitude compared to MMV007839 **1** and BH267.meta **2**.

### Cytotoxicity and In Vivo Activity

**Figure 5:**
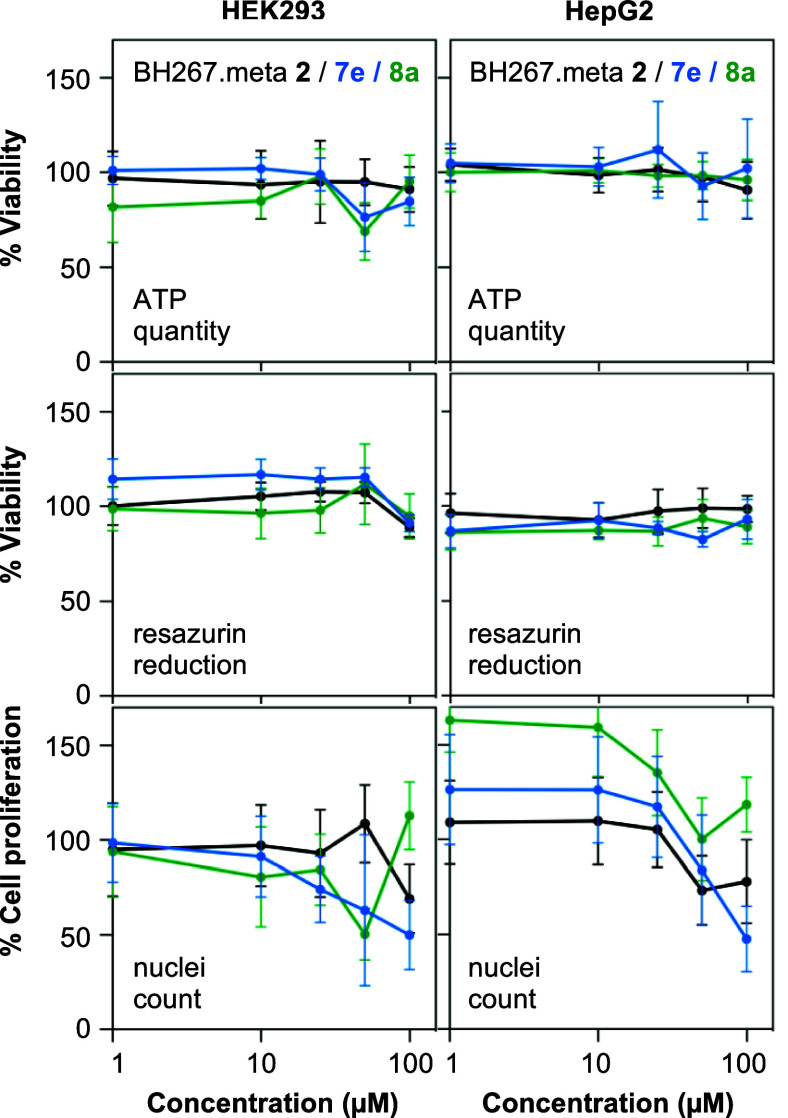
Cytotoxicity Assays. Shown is the effect of **7e** (blue) and **8a** (green) on HEK293 and HepG2 cells regarding viability (ATP levels, top; redox capability, center), and proliferation (bottom). BH267.meta **2** served as a reference (black). Error bars denote SEM (*n* = 3).

The high in vitro activity prompted us to assay representative *p*-amino and *p*-anilide-substituted BH267.meta **2** variants in vivo using mouse models. We selected **7e** (*n*-propylamine) and **8a** (acetanilide) for an initial analysis of their cytotoxicity toward HEK293 and HepG2 cells at concentrations up to 100 μM. Cell viability turned out to be unaffected over the full concentration range as determined by ATP levels (Figure 5, top) and redox capability of the cytosol (Figure 5, center). The nuclei count indicating cell proliferation started to decline slightly at concentrations above 10 μM (Figure 5, bottom). In relation to the in vitro activity of the compounds this gives a large safety margin of about 3 orders of magnitude.

**Figure 6:**
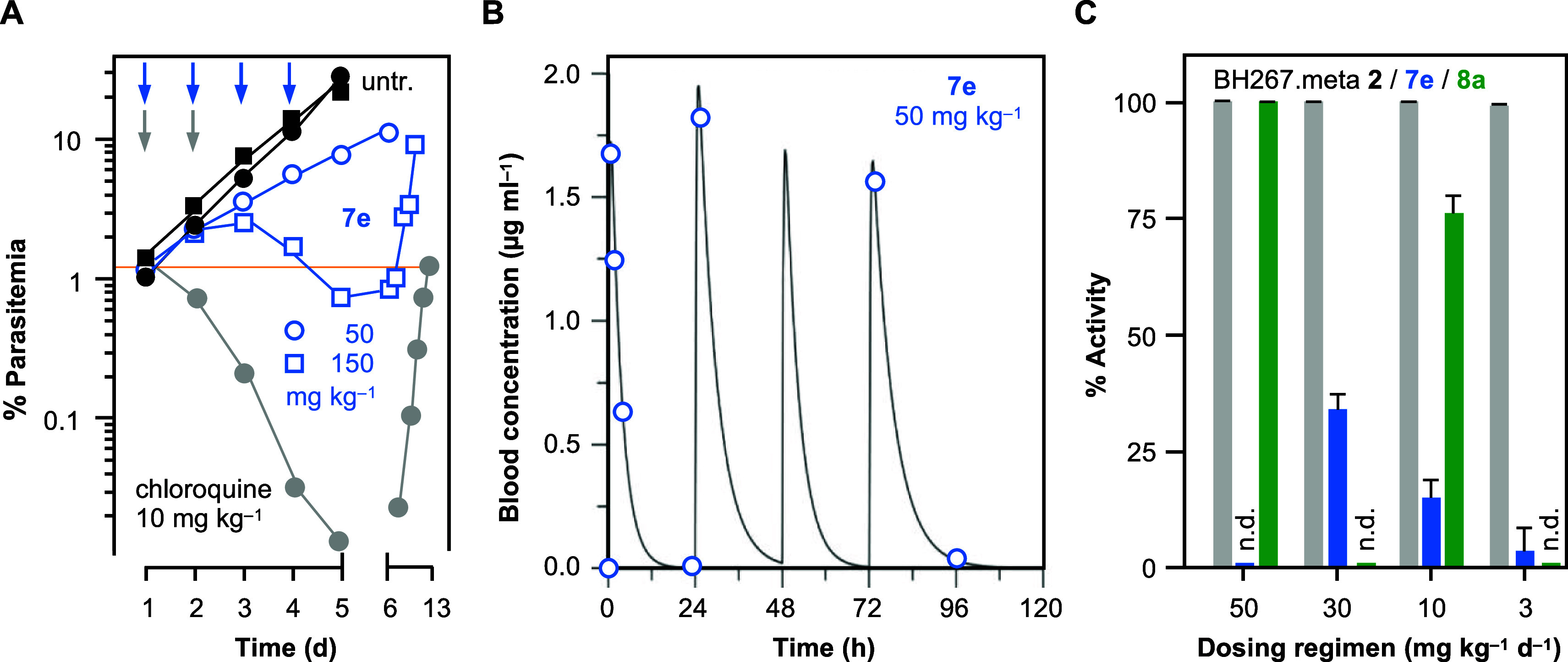
In vivo activity of **7e** and **8a**. (A) *P. falciparum* 3D7-infected SCID mice were left untreated (black), treated with 50 or 150 mg kg^–1^ of **7e** on four consecutive days (blue, arrows), or with 2 × 10 mg kg^–1^ chloroquine (gray). The orange line indicates the starting parasitemia. (B) Shown are the blood levels (circles) and the modeled profile during treatment with 50 mg kg^–1^ of **7e** on four consecutive days. (C) *P. berghei*-infected mice received oral doses of **7e** (blue) or **8a** (green) on four consecutive days. Activity was determined from parasitemia relative to untreated animals (S.E.M., *n* = 3). BH267.meta **2** data (gray) are shown for comparison.^10^

The in vivo activity of **7e** (the most potent in vitro compound), was assessed in comparison to chloroquine in the *P. falciparum* 3D7 SCID mouse model^16,17^ following oral administration of 50 or 150 mg kg^–1^ d^–1^ on four consecutive days to one animal each (Figure 6A). Here, only the 150 mg kg^–1^ day^–1^ dosing regimen decreased the parasite load below the starting parasitemia (Figure 6A, orange line). High blood concentrations of **7e** close to 2 μg mL^–1^ indicated good bioavailability, which was followed by a rapid decline of available compound after each of the four administrations of 50 mg kg^–1^ (Figure 6B). A short 2–4 h half-life of **7e** was determined from the modeled profile.

Efficacy in the *P. berghei* model was determined for both, **7e** and **8a**, by 4 day Peters’ test^18,19^ after oral administration of 50, 30, 10, or 3 mg kg^–1^ day^–1^ for four consecutive days. Here, **7e** reached 34.2%, 15.4%, and 3.5% activity at 30, 10, and 3 mg kg^–1^ (Figure 6C, blue bars). **8a** exhibited higher activity clearing 99.7% of the parasites at 50 mg kg^–1^, and 76.0% at 10 mg kg^–1^ (Figure 6C, green bars). Previously, the reference compound BH267.meta **2** eliminated >99.7% of the parasites at all dosing regimens^10^ (Figure 6C, gray bars).

We figured that the lower in vivo efficacy of **8a** and in particular of **7e** compared to BH267.meta **2** despite a 10–20 times higher in vitro activity may be related to pharmacokinetic properties in connection with the newly introduced amine or anilide moieties. Determination of log *D* at pH 7.4 showed that **7e** is considerably more lipophilic than **2** and **8a** due to the *n*-propyl chain (Table 4). Consequently, the plasma protein binding of **7e** was found to be strongly increased (Table 4) resulting in an about 5 times lower free compound concentration of 1.54% compared to 6.31% for **8a**, and 8.76% for **2**. Since the SCID mouse blood concentration profile of **7e** (Figure 6B) indicated rapid elimination or metabolism, we determined clearance and half-life by exposure to isolated human liver microsomes (Table 4). The secondary amine **7e** was metabolized exceedingly fast, i.e. by up to 2 orders of magnitude faster rates, while clearance of the anilide **8a** was more moderate, yet still occurred 2.7 times faster than that of **2**. The apparent correlation between the in vivo activity and microsomal clearance indicates that metabolic stability is a major determinant of the in vivo efficacy of the new PfFNT inhibitors.

## Discussion and Conclusions

The previous generations of PfFNT inhibitors, such as the initial library hit MMV007839 **1** or the later G107S resistance-circumventing BH267.meta **2**, already showed high-affinity binding^20^ (Table 1), which was restricted to the inner pocket above Tyr31 (Figure 1B). Their linear structure and mostly lipophilic surface in combination with the vinylogous acid moiety properly match the shape and dimension of the available space within the substrate transport path up to the center of the protein.^11^

This study shows that the cytosolic vestibule offers additional sites for interaction. New inhibitors carrying a nitrogen atom in the p-position of the aromatic ring, in the form of a secondary amine or anilide (Tables 2 and 3), consistently displayed a gain in binding affinity, which is in accordance with additional interactions, most likely hydrogen bonds to the phenol side chain of Tyr31 and the backbone carbonyl of Ser102. Elongation of the N-substituent yielded increasing activity for compounds with linear or flat alkyl moieties up to 3–4 carbons; further or more bulky extensions decreased the activity again. This finding corresponds well with the presence of a flat 4 Å long narrowing next to Tyr31 (Figure 1B). Occupying this region by a p-substituent may shield off bulk water from the inner binding site and additionally enhance the gain in affinity from polar interactions of the vinylogous acid and amine/anilide moieties. The rapidly increasing shallowness of the vestibule below Tyr31 exposes inhibitors to the aqueous solvent causing a penalty for larger hydrophobic carbohydrate moieties.^21^ Interestingly, the nearby resistance-conveying G107S mutation site of PfFNT^4^ (Figure 2) can differentially affect the activity of compounds with p-substitution. Reduction in activity was seen for the anilide **8a** while the *n*-propylamine of **7e** was well tolerated. This may be an effect on the positioning of the Tyr31 side chain.

The increase by 1 order of magnitude in the in vitro activity against *P. falciparum* 3D7 parasites was the major finding of this work. While the target affinity expressed as *K*_*i*_ was derived by direct titration of free solubilized PfFNT protein with the inhibitors, target binding in the in vitro situation is characterized by (i) the albumin-bound vs free compound equilibrium,^22^ and (ii) rates of transmembrane diffusion of the compound into the parasite.^23,24^

**Figure 7:**
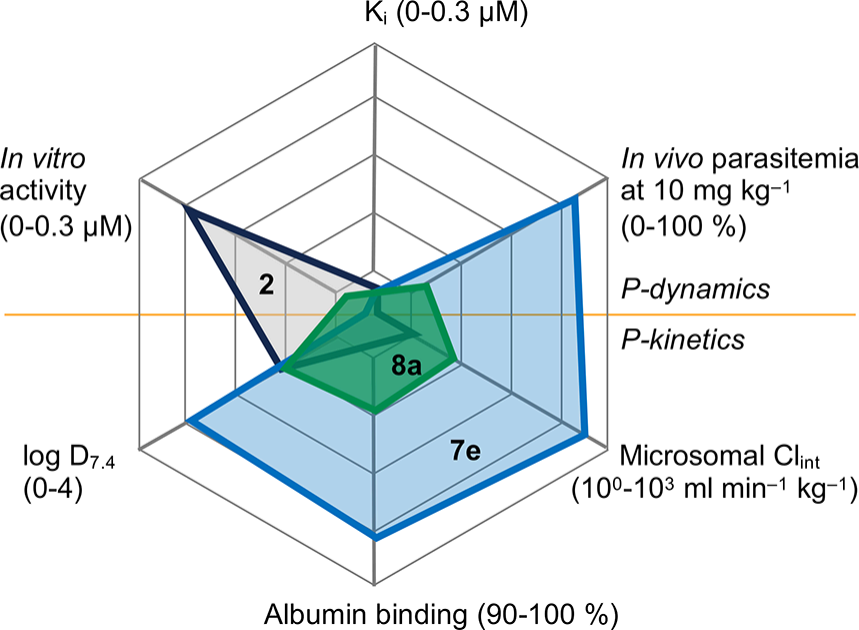
Comparison of pharmacodynamic and pharmacokinetic properties. Data from this study are shown for compounds **2** (gray), **7e** (blue), and **8a** (green). The scaling of the axes is indicated at the edges.

Both processes depend on the lipophilicity of the compound of interest.^25^ Higher lipophilicity enhances plasma protein binding and limits the free concentration. Lipophilic weak acids are particularly prone to adhere to plasma proteins^26^ as also seen here by >90% albumin binding of the compounds **2**, **7e**, and **8a** (Table 4, Figure 7). At the same time, higher lipophilicity promotes membrane penetration, while a too large molecule size can impede drug uptake. Such parameters need to be balanced for optimal in vitro activity.^27^ The most active compounds of this study in particular **7e** (Table 2) but also the anilide **8a** (Table 3) combine enhanced target binding with suitable in vitro properties in a favorable way leading to 10–20 times lower in vitro EC_50_ concentrations than the former **2** (Figure 7).

**Table 4:** Lipophilicity, Albumin Binding, and Metabolic Stability of PfFNT Inhibitors

	lipophilicity	albumin binding	human liver microsomes	
	(log *D*_7.4_)	(%)	Cl_int_ (mL min^–1^ kg^–1^)	*t*_1/2_ (min)
**2**	1.75	91.24	3.98	348
**7e**	3.18	98.46	462.00	3
**8a**	1.70	93.69	10.64	130

For adequate in vivo activity, **7e** fulfils the requirements of high bioavailability and location at the target site, i.e. the blood plasma (Figure 6B). However, rapid metabolism resulted in a too short half-life for an effective elimination of the parasites (Figure 7). A typical metabolism reaction of secondary amines is cytochrome P450-dependent oxidative *N*-dealkylation leading to the primary amine,^28^ i.e. in the case of **7e** an aniline moiety. The respective compound **7b** was analyzed in this study and showed at least 3 times lower in vitro activity (Table 2, Figure 4A). Anilines were shown to become N-hydroxylated by cytochrome P450 isoforms increasing polarity and elimination, and to give rise to toxic metabolites in subsequent activation pathways.^29^ The anilide **8a** exhibited higher in vivo efficacy than **7e** despite considerably lower in vitro activity of 49.8 vs 14.8 nM (Figure 4B). This is in line with the higher metabolic stability of amide vs amine bonds in general, and higher free fraction due to lower albumin binding, which were also observed here (Table 4, Figure 7).

In conclusion, the data of this study show that the in vitro activity of PfFNT inhibitors can be increased by 1 order of magnitude by addressing residues of the cytosolic vestibule via p-substitution of the aromatic ring. In order to translate this effect to the in vivo situation, the linking moieties require stabilization against metabolic conversion.

## Experimental Section

### Materials and Methods

#### Fluorescence Cross-Correlation Spectroscopy

FCCS measurements were conducted and analyzed as before.^20,30^ Briefly, membranes of stably transfected cells expressing PfFNT with a C-terminal GFP (green) were solubilized. BH267.meta **2** fused with DY647 (red) was used as a tracer to monitor the binding of compounds in a competition assay. Assays were done in 384-well glass bottom plates (cat. no. PS96B-G175, SWISSCI, Switzerland) using a plate reader (Insight, Evotec Technologies, Germany) fitted with a U-Apo300 40× water immersion lens, NA 1.15 (Olympus, Japan). Excitation was done with an argon-ion laser (488 nm) and a helium–neon laser (633 nm). Fluorescence fluctuations were recorded 10 times for 5 s per sample and fitted using FCS+ plus Analyze (PerkinElmer software). Initially, IC_50_ values were determined from the concentration of the dual labeled complex titrated against a competitor in a range of 100 pM to 100 μM (Figure S1). Then, *K*_*i*_ values were calculated using the Cheng Prusoff equation^31^ based on the previously determined K_D_ value for the BH267.meta-DY647 tracer.^20^

#### Yeast-Based PfFNT Transport Assays

Lactate transport functionality of PfFNT was assayed as described before.^4^ Briefly, *Saccharomyces cerevisiae* W303-1A jen1Δ*a*dy2Δ*c*ells^32^ (MATa, can1-100, ade2-loc, his3-11-15, leu2-3,-112, trp1-1-1, ura3-1, jen1::kanMX4, ady2::hphMX4) kindly provided by M. Casal expressing PfFNT was grown at 29 °C to an OD_600_ of 1 (±0.1). Harvested cells were resuspended to an OD_600_ of 50 (±5) in 50 mM HEPES/Tris, pH 6.8 (±0.1). 10–15 min prior to the assay, 80 μL of yeast suspension were added to 1 μL of DMSO with or without inhibitor in a 1.5 mL reaction tube at 18 °C. Twenty μL of l-lactate solution spiked with [1-^14^C]-l-lactate (Hartmann Analytic) was added yielding a 1 mM inward gradient with 0.04 μCi label. Transport was stopped after 30 s by abrupt dilution with 1 mL of ice-cold water, vacuum filtration via GF/C filter membranes (Whatman), and washing with 7 mL of ice-cold water. The radio label was analyzed by liquid scintillation counting in 3 mL of scintillation cocktail (Rotiszint eco plus, Carl Roth) using a Packard TriCarb 2900 TR (PerkinElmer Inc.).

#### *Plasmodium falciparum* Cultivation and Growth Assays

*P. falciparum* 3D7 parasites were grown in RPMI 1640 medium supplemented with 0.5% albumax according to standard procedures.^33^ For determination of in vitro drug susceptibility, asynchronous parasites (adjusted to 0.8% starting parasitemia) were exposed to different concentrations of the respective PfFNT inhibitor for 48 h with one medium change and inhibitor replenishment after 24 h. The parasitemia was measured using an LSR II FACS (BD Biosciences) as described.^34,35^ Growth was assessed as % of untreated control. EC_50_ curves were plotted using Graph Pad Prism.

#### Cytotoxicity Assays

HEK293T and HepG2 cells were precultured in Dulbecco’s modified Eagle medium with 10% fetal bovine serum (FBS, Gibco, #10270106) in 96-well plates (Sarstedt, TC-treated, #83.3924) at a density of 20,000 cells per well. For ATP quantification, commercial CellTiterGlo was used according to the manufacturer’s instructions. For the resazurin assay, the culture medium was removed, and the cells were incubated in 120 μL of a 66 μM resazurin solution in PBS (SigmaAldrich, #R7017) at 37 °C for 2 h, for fluorescence read-out (λ_ex_/λ_em_ = 544/590 nm) using a microplate reader (Spark, Tecan Group). Nuclei were counted by microscopy (ImageXpress MicroXL, Molecular Devices) after fixation with 4% paraformaldehyde in PBS, and 15 min incubation at room temperature with Hoechst33342. Quantification was done using the “Cell Proliferation High-Throughput” algorithm (MetaXpress Software, Molecular Devices). All values were normalized to vehicle control (DMSO).

#### In Vivo Activity of **7e** and **8a** Using Mouse Models

The animal experiments against *P. berghei* were carried out at the Swiss Tropical and Public Health Institute adhering to local and national regulations of laboratory animal welfare in Switzerland (awarded permission no. BL530). Protocols are regularly reviewed and revised following approval by the local authority (Amt für Lebensmittelsicherheit and Veterinärwesen Basel-Land). Female mice infected with GFP-expressing *P. berghei* (ANKA strain, donated by A. P. Waters and C. J. Janse, Leiden University, The Netherlands) were analyzed for in vivo efficacy of **7e** and **8a** as described previously.^18,19^ Parasitemia was determined using flow cytometry with a detection limit of 1 parasite in 1000 erythrocytes. Compound activity was calculated as the difference between the mean parasitemia for the control and treated groups (*n* = 3, each) expressed relative to the control group. **7e** and **8a** were suspended in 70/30 Tween 80/ethanol, diluted 10-fold with water and administered orally 4, 24, 48, and 72 h after infection. **7e** was analyzed at 30, 10, and 3 mg kg^–1^, **8a** at 50, and 10 mg kg^–1^. In vivo efficacy against *P. falciparum* was conducted at The Art of Discovery, TAD, Bizkaia, Spain, according to the previously described standard assay.^16,17^

#### Determination of Lipophilicity, Log *D*_7.4_

Lipophilicity was determined according to the OECD Guidelines for the Testing of Chemicals no. 177. Briefly, the test compounds plus 9 standards (4-acetylpyridine, acetanilide, acetophenone, methyl benzoate, ethyl benzoate, benzophenone, phenyl benzoate, diphenyl ether, triphenylamine) were separated by HPLC with UV detection at 220 nm (Waters HPLC 1525 with tunable absorbance detector 486) using an RP-8 column (Agilent Technologies ZORBAX Eclipse XDB-C8 4.6 × 150 mm, 5 μm) at 20 °C. Eluents A (20 mM ammonium formate, pH 7.4) and B (methanol) were used to generate a gradient profile of 80% A–100% B (0–35 min), 100% B (35–37 min), 100% B–80% A (37–42 min), and 80% A (42–55 min) at a flow rate of 0.8 mL min^–1^. The dead time was determined via the injection peak caused by the solvent. The log *D*_7.4_ values were calculated by correlating the capacity factors with the known values of the standards via polynomial fitting.

#### Microsomal Clearance, Half-Life, and Plasma Protein Binding

Clearance, half-life and plasma protein binding were assayed at TGC LifeScience, Kolkata, India, as described before.^36^ Test compounds were incubated in duplicate in 96-well plates at 1 μM concentration with pooled human liver microsomes in 0.1 M phosphate buffer, pH 7.4 (0.4 mg mL^–1^ final protein concentration; XenoTech, Lenexa, KS) in the presence and absence of 1 mM NADPH. Metabolism reactions were stopped by addition of 300 μL of ice-cold acetonitrile containing carbamazepine at 0.0236 μg mL^–1^ as an internal standard, collection and analysis of the supernatant by LC–MS/MS (Agilent Rapid Resolution HPLC, AB SCIEX 4500 MS). The relative loss of the test compound over time was used to determine the first order rate constant for calculation of the in vitro intrinsic clearance and half-life.^37^ Plasma protein binding was determined by ultracentrifugation from pooled human plasma spiked with 5 μM test compound. Aliquots were transferred immediately (total plasma concentration) or after 1 h at 37 °C (protein bound fraction) to ice-cold acetonitrile containing 0.0236 μg mL^–1^ carbamazepine as an internal standard, ultracentrifuged for 4 h at 37 °C (Beckman Optima L-80XP), and analyzed by LC–MS/MS (Agilent Rapid Resolution HPLC, AB SCIEX 4500 QTRAP MS).

#### Virtual Docking

Docking was done using Maestro 12.8 (release 2021-2, Schrödinger).^38^ The PfFNT structure^11^ (PBD# 7E27) was imported and processed using the Protein Preparation Wizard. For docking the standard ligand centered Induced Fit Docking protocol was used, providing flexibility for the ligand and the target protein binding pocket.^39^ Visualization was done using Chimera^40^ (Version1.16).

#### Statistical Analysis

Transport assays in yeast were done in triplicate biological replicates. At least nine technical replicates were measured around the IC_50_ value, concentrations with no or full inhibition were determined from at least three technical replicates. EC_50_-values were determined from 2 to 4 biological replicates. IC_50_ and EC_50_ curves were fitted by the Hill equation with variable slope (Graph Pad Prism 10). Error bars denote ± SEM (for *n* ≥ 3) or ± range (for *n* = 2). For the EC_50_-value of **7a** the error of the fitting of the curve is given. Cytotoxicity was determined from biological triplicates; error is shown as SEM.

## Chemistry

### General Procedure for Synthesis

If not described differently, the first step in the synthesis of compounds **7a**, **c**–**t** was done using 1 equiv 1-(6-chloropyridin-3-yl)ethan-1-one with 4 equiv of the respective amine in isopropanol at 120 °C for 1.5 h using a microwave (CEM Discover system). Subsequently, the solvent was evaporated, and the residue was dissolved in ethyl acetate, washed with brine, dried over Na_2_SO_4_, filtered and evaporated to dryness. The residue was purified by flash chromatography (Büchi Pure C-815) resulting in the respective 1-(6-(alkylamino)pyridin-3-yl)ethan-1-one. For compounds **3a**–**d**, **5a**–**b**, **5d**, **7a**, **7c**–**t**, a Claisen-type condensation was done with 1 equiv of the ketone and 1.1 equiv of the ester component in anhydrous THF. The reaction was started by adding 1.4 equiv of sodium methoxide (30% solution) at 0 °C, and stirred overnight at room temperature. The solvent was evaporated, and the residue (in acetic acid/water at 7:50) was extracted with ethyl acetate or DCM for purification by column or flash chromatography (Büchi Pure C-815) and/or recrystallization. All compounds (see Table S1 for SMILES list) exhibited >95% purity as determined by HPLC (Shimadzu Nexera CL and Waters HPLC 1525 with tunable absorbance detector 486; Figure S4). Structure analysis was done by mass spectrometry (LC–MS; Bruker Amazon SL), and nuclear magnetic resonance (Bruker Avance III 400).

### Compounds

#### 4,4-Difluoro-3-hydroxy-4-phenyl-1-(pyridin-3-yl)but-2-en-1-one **3a**

Applying the general procedure with ethyl-2,2-difluoro-2-phenylacetate (18 mmol) and 3-acetylpyridine (16 mmol) delivered the titled compound **3a** (3.82 g, 87%). ^1^H NMR (400 MHz, DMSO-*d*_6_): δ/ppm 9.23 (d, ^4^*J* = 2.0 Hz, 1H), 8.82 (dd, ^3^*J* = 4.8 Hz, ^4^*J* = 1.6 Hz, 1H), 8.42 (dt, ^3^*J* = 8.1 Hz, ^4^*J* = 2.0 Hz, 1H), 7.70 (m, 2H), 7.60 (m, 1H), 7.57 (m, 3H), 7.08 (s, 1H). ^13^C NMR (101 MHz, DMSO-*d*_6_): δ/ppm 206.5 (1C), 185.7 (1C), 181.9 (1C), 153.8 (1C), 148.6 (1C), 135.2 (1C), 132.6 (1C), 131.3 (1C), 129.0 (2C), 125.5 (2C), 124.0 (1C), 115.4 (1C), 93.3 (1C). MS (ESI negative) *m*/*z* (%): 274 (100) [M – H]^−^, 571 (46) [2M – 2H + Na]^−^.

#### 4,4,5,5,6,6,6-Heptafluoro-3-hydroxy-1-(pyridin-3-yl)hex-2-en-1-on **3b**

Applying the general procedure with ethyl heptafluorobutyrate (9 mmol) and 3-acetylpyridine (8 mmol) delivered the titled compound **3b** (2,15 g, 85%). ^1^H NMR (400 MHz, DMSO-*d*_6_): δ/ppm 9.22 (d, ^4^*J* = 1.5 Hz, 1H), 8.85 (dd, ^3^*J* = 4.9 Hz, ^4^*J* = 1.5 Hz, 1H), 8.48 (dt, ^3^*J* = 8.0 Hz, ^4^*J* = 1.5 Hz, 1H), 7.67 (dd, ^3^*J* = 8.0 Hz, ^3^*J* = 4.9 Hz, 1H), 6.86 (s, 1H). ^13^C NMR (101 MHz, DMSO-*d*_6_): δ/ppm 182.6 (1C), 174.6 (1C), 152.6 (1C), 148.0 (1C), 136.7 (1C), 130.4 (1C), 124.3 (1C), 94.6 (1C). MS (ESI negative) *m*/*z* (%): 316 (100) [M – H]^−^, 655 (10) [2M–2H + Na]^−^.

#### 4,4,5,5-Tetrafluoro-3-hydroxy-5-methoxy-1-(pyridin-3-yl)pent-2-en-1-one **3c**.

Applying the general procedure with methyl-2,2,3,3-tetrafluoro-3-methoxypropanoate (4.5 mmol) and 3-acetylpyridine (4.5 mmol) delivered the titled compound **3c** (462 mg, 37%). ^1^H NMR (400 MHz, DMSO-*d*_6_): δ/ppm 9.22 (d, ^4^*J* = 1.9 Hz, 1H), 8.83 (dd, ^3^*J* = 4.8 Hz, ^4^*J* = 1.6 Hz, 1H), 8.43 (dt, ^3^*J* = 8.1 Hz, ^4^*J* = 1.9 Hz, 1H), 7.61 (m, 1H), 6.93 (s, 1H), 3.71 (s, 3H). ^13^C NMR (101 MHz, DMSO-*d*_6_): δ/ppm 182.2 (1C), 179.2 (1C), 154.0 (1C), 148.7 (1C), 135.5 (1C), 128.7 (1C), 124.1 (1C), 117.6 (1C), 108.8 (1C), 94.8 (1C), 52.0 (1C). MS (ESI negative) *m*/*z* (%): 278 (100) [M – H]^−^, 579 (9) [2M – 2H + Na]^−^.

#### 5-Chloro-4,4,5,5-tetrafluoro-3-hydroxy-1-(pyridin-3-yl)pent-2-en-1-one **3d**

Applying the general procedure with ethyl-3-chloro-2,2,3,3-tetrafluoropropionate (2.57 mmol) and 3-acetylpyridine (2.29 mmol) delivered the titled compound **3d** (450 mg, 62%). ^1^H NMR (400 MHz, DMSO-*d*_6_): δ/ppm 9.23 (m, 1H), 8.85 (dd, ^3^*J* = 4.9 Hz, ^4^*J* = 1.5 Hz, 1H), 8.47 (d, ^3^*J* = 7.8 Hz, 1H), 7.65 (dd, ^3^*J* = 7.8 Hz, ^3^*J* = 4.9 Hz, 1H), 6.96 (s, 1H). ^13^C NMR (101 MHz, DMSO-*d*_6_): δ/ppm 182.5 (1C), 176.2 (1C), 153.2 (1C), 148.4 (1C), 136.2 (1C), 129.6 (1C), 124.2 (1C), 122.0 (1C), 108.8 (1C), 95.0 (1C). MS (ESI negative) *m*/*z* (%): 282 (100) [M – H]^−^, 587 (39) [2M – 2H + Na]^−^.

#### 3-[5-(1,1,2,2,2-Pentafluoroethyl)-1*H*-1,2,4-triazol-3-yl]pyridine **4a**

For the synthesis,^41^ hydrazine monohydrate (7 mmol, dissolved in ethanol) and ethyl pentafluoropropionate (7 mmol) were mixed at 0 °C, and stirred for 90 min obtaining pentafluoropropionhydrazid. Sodium methoxide was added to a suspension of nicotinamidine–HCl (1.5 g in ethanol), stirred at room temperature for 1 h and filtered. Pentafluoropropionhydrazid was added to the filtrate, and stirred at room temperature under argon atmosphere for 4 d. A 1:1 mixture of diethyl ether and cyclohexan was added, the solvent was decanted, and evaporated. The precipitate was treated with 12 mL 3 N NaOH solution under reflux for 1.5 h. The suspension was filtered and the solid was purified by column chromatography to give the titled compound **4a** (122 mg, 7%). ^1^H NMR (400 MHz, DMSO-*d*_6_): δ/ppm 15.67 (br s, 1H), 9.21 (dd, ^4^*J* = 2.2 Hz, ^4^*J* = 0.7 Hz, 1H), 8.76 (dd, ^3^*J* = 4.8 Hz, ^4^*J* = 1.7 Hz, 1H), 8.39 (dtd, ^3^*J* = 8.0 Hz, ^4^*J* = 1.7 Hz, ^4^*J* = 0.5 Hz, 1H), 7.65 (ddd, ^3^*J* = 8.0 Hz, ^3^*J* = 4.8 Hz, ^4^*J* = 0.8 Hz, 1H). ^13^C NMR (101 MHz, DMSO-*d*_6_): δ/ppm 158.5 (1C), 154.5 (1C), 151.6 (1C), 147.3 (1C), 134.4 (1C), 124.3 (1C), 122.4 (1C), 118.3 (1C), 109.2 (1C). MS (ESI negative) *m*/*z* (%): 263 (100) [M – H]^−^, 527 (83) [2M – H]^−^, 549 (4) [2M – 2H + Na]^−^.

#### 3-[5-(1,1,2,2,2-Pentafluoroethyl)-1*H*-pyrazol-3-yl]pyridine **4b**

4,4,5,5,5-Pentafluoro-3-hydroxy-1-(pyridin-3-yl)pent-2-en-1-one, BH267.meta **2**, was treated with hydrazine monohydrate in THF to the titled compound **4b**. ^1^H NMR (400 MHz, DMSO-*d*_6_): δ/ppm 14.35 (br s, 1H), 9.07 (d, ^4^*J* = 2.0 Hz, 1H), 8.61 (dd, ^3^*J* = 4.7 Hz, ^4^*J* = 1.4 Hz, 1H), 8.22 (dt, ^3^*J* = 8.0 Hz, ^4^*J* = 1.9 Hz, 1H), 7.65 (dd, ^3^*J* = 8.0 Hz, ^3^*J* = 4.8 Hz, 1H), 7.37 (s, 1H). ^13^C NMR (101 MHz, DMSO-*d*_6_): δ/ppm 149.8 (1C), 146.7 (1C), 141.5 (1C), 140.7 (1C), 132.9 (1C), 124.1 (1C), 124.0 (1C), 118.6 (1C), 110.9 (1C), 102.9 (1C). MS (ESI negative) *m*/*z* (%): 262 (100) [M – H]^−^, 525 (50) [2M – H]^−^.

#### 4,4,5,5,5-Pentafluoro-1-(furan-2-yl)-3-hydroxypent-2-en-1-one **5a**

Applying the general procedure with ethyl pentafluoropropionate (18 mmol) and 2-acetylfuran (16 mmol), and distillation delivered the titled compound **5a** (250 mg, 6%). ^1^H NMR (400 MHz, CDCl_3_): δ/ppm 14.64 (br s, 1H), 7.69 (m, 1H), 7.36 (d, *J* = 3.6 Hz, 1H), 6.65 (dd, *J* = 3.6 Hz, *J* = 1.7 Hz, 1H), 6.72 (s, 1H). ^13^C NMR (101 MHz, CDCl_3_): δ/ppm 176.5 (1C), 175.2 (1C), 149.0 (1C), 148.2 (1C), 119.0 (1C), 118.3 (1C), 113.6 (1C), 107.9 (1C), 94.3 (1C). MS (ESI negative) *m*/*z* (%): 255 (100) [M – H]^−^.

#### 4,4,5,5,5-Pentafluoro-1-(furan-3-yl)-3-hydroxypent-2-en-1-one **5b**

Applying the general procedure with ethyl pentafluoropropionate (5.1 mmol) and 3-acetylfuran (4.5 mmol), and distillation delivered the titled compound **5b** (77 mg, 7%). ^1^H NMR (400 MHz, CDCl_3_): δ/ppm 14.88 (br s), 8.15 (m, 1H), 7.53 (m, 1H), 6.76 (dd, *J* = 1.9 Hz, *J* = 0.7 Hz, 1H), 6.27 (s, 1H). ^13^C NMR (101 MHz, CDCl_3_): δ/ppm 181.5 (1C), 177.3 (1C), 147.6 (1C), 145.2 (1C), 123.4 (1C), 118.3 (1C), 108.1 (1C), 107.8 (1C), 94.9 (1C). MS (ESI negative) *m*/*z* (%): 255 (100) [M – H]^−^.

#### 4,4,5,5,5-Pentafluoro-3-hydroxy-1-(thiophen-2-yl)pent-2-en-1-one **5c**

The compound was purchased (purity 95.8%; abcr GmbH, Karlsruhe). ^1^H NMR (400 MHz, DMSO-*d*_6_): δ/ppm 12.20 (s, 1H), 8.38 (dd, *J* = 3.9 Hz, *J* = 0.9 Hz, 1H), 8.21 (dd, *J* = 4.9 Hz, *J* = 0.9 Hz, 1H), 7.33 (dd, *J* = 4.9 Hz, *J* = 3.9 Hz, 1H), 7.01 (s, 1H). ^13^C NMR (101 MHz, DMSO-*d*_6_): δ/ppm 182.5 (1C), 168.1 (1C), 139.5 (1C), 137.8 (1C), 135.4 (1C), 129.6 (1C), 118.0 (1C), 107.8 (1C), 95.8 (1C). MS (ESI negative) *m*/*z* (%): 271 (100) [M – H]^−^.

#### 4,4,5,5,5-Pentafluoro-3-hydroxy-1-(thiophen-3-yl)pent-2-en-1-one **5d**

Applying the general procedure with ethyl pentafluoropropionate (15 mmol) and 3-acetylthiophene (12.5 mmol), and distillation delivered the titled compound **5d** (2.2 g, 65%). ^1^H NMR (400 MHz, DMSO-*d*_6_): δ/ppm 12.40 (s, 1H), 8.83 (dd, *J* = 2.8 Hz, 1.4 Hz, 1H), 7.75 (m, 2H), 6.94 (s, 1H). ^13^C NMR (101 MHz, DMSO-*d*_6_): δ/ppm 180.8 (1C), 174.6 (1C), 136.5 (1C), 135.5 (1C), 128.8 (1C), 126.2 (1C), 118.0 (1C), 107.5 (1C), 95.0 (1C). MS (ESI negative) *m*/*z* (%): 271 (100) [M – H]^−^.

#### 4,4,5,5,5-Pentafluoro-3-hydroxy-1-(3-hydroxyphenyl)pent-2-en-1-one **6**

Lithium hydride (42.8 mmol) was suspended in anhydrous THF, then ethyl pentafluoropropionate (15 mmol) and 3-hydroxyacetophenon (12.5 mmol) were added dropwise. The reaction was stirred at 50 °C for 3 h following purification according to the general procedure delivering the titled compound **6** (2.36 g, 67%). ^1^H NMR (400 MHz, DMSO-*d*_6_): δ/ppm 9.97 (s, 1H), 7.58 (d, ^3^*J* = 8.0 Hz, 1H), 7.44 (m, 1H), 7.38 (t, ^3^*J* = 8.0 Hz, 1H), 7.12 (dd, ^3^*J* = 8.0 Hz, ^4^*J* = 2.4 Hz, 1H), 6.92 (s, 1H). ^13^C NMR (101 MHz, DMSO-*d*_6_): δ/ppm 185.6 (1C), 175.5 (1C), 157.9 (1C), 133.7 (1C), 130.3 (1C), 121.8 (1C), 119.1 (1C), 117.9 (1C), 114.0 (1C), 107.5 (1C), 94.2 (1C). MS (ESI negative) *m*/*z* (%): 281 (100) [M – H]^−^, 563 (16) [2M – H]^−^.

#### 1-[6-(Benzylamino)pyridin-3-yl]-4,4,5,5,5-pentafluoro-3-hydroxypent-2-en-1-one **7a**

Applying the general procedure with benzylamine at 150 °C resulted in 1-(6-(benzylamino)pyridin-3-yl)ethan-1-one (333 mg, 49%). Then 1-(6-(benzylamino)pyridin-3-yl)ethan-1-one (1.4 mmol) was reacted with ethyl pentafluoropropionate (1.6 mmol) delivering after sublimation the titled compound **7a** (48 mg, 9%). ^1^H NMR (400 MHz, CDCl_3_): δ/ppm 8.70 (d, ^4^*J* = 1.7 Hz, 1H), 7.95 (dd, ^3^*J* = 8.9 Hz, ^4^*J* = 2.4 Hz, 1H), 7.37 (m, 5H), 6.45 (d, ^3^*J* = 8.9 Hz, 1H), 6.45 (s, 1H) 5.88 (s, 1H), 4.62 (d, ^3^*J* = 8.9 Hz, 2H). ^13^C NMR (101 MHz, CDCl_3_): δ/ppm 185.2 (1C), 176.1 (1C), 161.3 (1C), 150.7 (1C), 137.8 (1C), 136.9 (1C), 129.1 (1C), 128.0 (2C), 127.6 (2C), 118.5 (1C), 118.4 (1C), 108.0 (1C), 106.9 (1C), 92.4 (1C), 46.3 (1C). MS (ESI negative) *m*/*z* (%): 371 (100) [M – H]^−^, 743 (21) [2M – H]^−^.

#### 1-(6-Aminopyridin-3-yl)-4,4,5,5,5-pentafluoro-3-hydroxypent-2-en-1-one **7b**

1-(6-Chloropyridin-3-yl)ethan-1-one (25.7 mmol) was mixed with 75 mL ammonium hydroxide (33%) and heated to 160 °C for 5 h. After cooling, the solution was neutralized using HCl (25%), and extracted with ethyl acetate giving 1-(6-aminopyridin-3-yl)ethan-1-one (3.45 g, 99%). 7.2 mmol of the product were reacted at room temperature with di-*tert*-butyl dicarbonate in the presence of DMAP (80 mg) and triethylamine (10.8 mmol) in DCM. Flash-chromatography isolated *tert*-butyl (5-acetylpyridin-2-yl)carbamate (790 mg, 46%), of which 11.8 mmol were reacted in a Claisen-type condensation according to the general procedure. to *tert*-butyl-(5-(4,4,5,5,5-pentafluoro-3-hydroxypent-2-enoyl)pyridin-2-yl)carbamate (3.49 g, 77%). The protection group was removed from 1.48 mmol of the product with TFA in DCM at 0 °C delivering the titled compound **7b** (415 mg, 99%, total yield: 35%). ^1^H NMR (400 MHz, DMSO-*d*_6_): δ/ppm 8.81 (d, ^4^*J* = 2.4 Hz, 1H), 8.04 (dd, ^3^*J* = 9.0 Hz, ^4^*J* = 2.5 Hz, 1H), 7.46 (s, 2H), 6.85 (s, 1H), 6.54 (d, ^3^*J* = 9.0, 1H). ^13^C NMR (101 MHz, DMSO-*d*_6_): δ/ppm 185.3 (1C), 173.0 (1C), 163.2 (1C), 151.9 (1C), 136.7 (1C), 118.1 (1C), 116.2 (1C), 108.1 (1C), 107.7 (1C), 92.3 (1C). MS (ESI negative) *m*/*z* (%): 281 (100) [M – H]^−^, 585 (12) [2M – 2H + Na]^−^.

#### 4,4,5,5,5-Pentafluoro-3-hydroxy-1-(6-(methylamino)pyridin-3-yl)pent-2-en-1-one **7c**

Applying the general procedure with 40% methylamine solution resulted in 1-(6-(methylamino)pyridin-3-yl)ethan-1-one (596 mg, 99%). 3.7 mmol of the product were reacted with 4.2 mmol ethyl pentafluoropropionate delivering the titled compound **7c** (529 mg, 48%). ^1^H NMR (400 MHz, DMSO-*d*_6_): δ/ppm 8.88 (m, 1H), 7.98 (m, 2H), 6.86 (s, 1H), 6.45 (d, ^3^*J* = 9.1 Hz, 1H), 2.90 (d, *J* = 3.8, 3H). ^13^C NMR (101 MHz, DMSO-*d*_6_): δ/ppm 185.5 (1C), 173.1 (1C), 162.2 (1C), 152.1 (1C), 135.2 (1C), 117.9 (1C), 115.7 (1C), 109.3 (1C), 107.7 (1C), 92.4 (1C), 27.7 (1C). MS (ESI negative) *m*/*z* (%): 295 (100) [M – H]^−^, 613 (15) [2M – 2H + Na]^−^.

#### 1-(6-(Ethylamino)pyridin-3-yl)-4,4,5,5,5-pentafluoro-3-hydroxypent-2-en-1-one **7d**

Applying the general procedure with 70% methylamine solution resulted in 1-(6-(ethylamino)pyridin-3-yl)ethan-1-one (993 mg, 91%). 2.9 mmol of the product were reacted with 3.4 mmol ethyl pentafluoropropionate delivering the titled compound **7d** (687 mg, 76%). ^1^H NMR (400 MHz, DMSO-*d*_6_): δ/ppm 8.87 (d, ^4^*J* = 2.3 Hz, 1H), 8.00 (m, 2H), 6.86 (s, 1H), 6.55 (d, ^3^*J* = 9.1 Hz 1H), 3.40 (m, 2H), 1.16 (t, ^3^*J* = 7.2 Hz, 3H). ^13^C NMR (101 MHz, DMSO-*d*_6_): δ/ppm 185.4 (1C), 173.1 (1C), 161.6 (1C), 152.2 (1C), 135.4 (1C), 118.1 (1C), 115.6 (1C), 109.2 (1C), 107.6 (1C), 92.3 (1C), 35.5 (1C), 14.3 (1C). MS (ESI negative) *m*/*z* (%): 309 (100) [M – H]^−^, 619 (12) [2M – H]^−^, 641 (15) [2M – 2H + Na]^−^.

#### 4,4,5,5,5-Pentafluoro-3-hydroxy-1-(6-(propylamino)pyridin-3-yl)pent-2-en-1-one **7e**.

Applying the general procedure with *n*-propylamine resulted in 1-(6-(propylamino)pyridin-3-yl)ethan-1-one (1,08 g, 76%). 2.8 mmol of the product were reacted with 3.2 mmol ethyl pentafluoropropionate delivering the titled compound **7e** (661 mg, 73%). ^1^H NMR (400 MHz, CDCl_3_): δ/ppm 8.72 (d, ^4^*J* = 2.2 Hz, 1H), 7.95 (dd, ^3^*J* = 8.9 Hz, ^4^*J* = 2.2 Hz, 1H), 6.46 (s, 1H), 6.42 (d, ^3^*J* = 8.9 Hz, 1H), 5.32 (s, 1H), 3.35 (q, 2H), 1.69 (sxt, ^3^*J* = 7.3, 2H), 1.02 (t, ^3^*J* = 7.4, 3H). ^13^C NMR (101 MHz, CDCl_3_): δ/ppm 185.4 (1C), 173.1 (1C), 161.8 (1C), 152.2 (1C), 135.3 (1C), 118.1 (1C), 115.6 (1C), 109.2 (1C), 107.6 (1C), 92.3 (1C), 42.4 (1C), 22.0 (1C), 11.4 (1C). MS (ESI negative) *m*/*z* (%): 323 (100) [M – H]^−^, 647 (9) [2M – H]^−^, 669 (7) [2M – 2H + Na]^−^.

#### 1-(6-(Butylamino)pyridin-3-yl)-4,4,5,5,5-pentafluoro-3-hydroxypent-2-en-1-one **7f**

Applying the general procedure with *n*-butylamine resulted in 1-(6-(butylamino)pyridin-3-yl)ethan-1-one (360 mg, 47%). 1.8 mmol of the product were reacted with 2.0 mmol ethyl pentafluoropropionate delivering the titled compound **7f** (190 mg, 32%). ^1^H NMR (400 MHz, CDCl_3_): δ/ppm 8.70 (d, ^4^*J* = 2.2 Hz, 1H), 7.97 (dd, ^3^*J* = 9.0 Hz, ^4^*J* = 1.6 Hz, 1H), 6.45 (d, ^3^*J* = 9.0 Hz, 1H), 6.45 (s, 1H), 5.72 (s, 1H), 3.38 (m, 2H), 1.65 (qui, ^3^*J* = 7.2, 2H), 1.44 (sxt, ^3^*J* = 7.5, 2H), 0.97 (t, ^3^*J* = 7.3, 3H). ^13^C NMR (101 MHz, CDCl_3_): δ/ppm 185.1 (1C), 175.8 (1C), 161.2 (1C), 150.2 (1C), 137.0 (1C), 118.4 (1C), 118.0 (1C), 108.2 (1C), 106.5 (1C), 92.3 (1C), 42.1 (1C), 31.4 (1C), 20.2 (1C), 13.9 (1C). MS (ESI negative) *m*/*z* (%): 337 (100) [M – H]^−^.

#### 4,4,5,5,5-Pentafluoro-3-hydroxy-1-(6-(pentylamino)pyridin-3-yl)pent-2-en-1-one **7g**

Applying the general procedure with *n*-pentylamine resulted in 1-(6-(pentylamino)pyridin-3-yl)ethan-1-one (1,53 g, 74%). 3.6 mmol of the product reacted with 4.0 mmol ethyl pentafluoropropionate delivering the titled compound **7g** (965 mg, 75%). ^1^H NMR (400 MHz, DMSO-*d*_6_): δ/ppm 8.86 (d, ^4^*J* = 2.2 Hz, 1H), 8.00 (m, 2H), 6.85 (s, 1H), 6.56 (d, ^3^*J* = 9.0 Hz, 1H), 3.37 (m, 2H), 1.54 (m, 2H), 1.30 (m, 4H), 0.87 (m, 3H). ^13^C NMR (101 MHz, DMSO-*d*_6_): δ/ppm 185.4 (1C), 173.1 (1C), 161.7 (1C), 152.3 (1C), 135.2 (1C), 117.9 (1C), 115.5 (1C), 109.2 (1C), 107.6 (1C), 92.3 (1C), 40.6 (1C), 28.7 (1C), 28.4 (1), 21.9 (1C), 13.9 (1C). MS (ESI negative) *m*/*z* (%): 351 (100) [M – H]^−^, 725 (42) [2M – 2H + Na]^−^.

#### 4,4,5,5,5-Pentafluoro-3-hydroxy-1-(6-((3-methoxypropyl)amino)pyridin-3-yl)pent-2-en-1-one **7h**

Applying the general procedure with 3-methoxypropylamine resulted in 1-(6-((3-methoxypropyl)amino)pyridin-3-yl)ethan-1-one (1.03 g, 82%). 2.4 mmol of the product were reacted with 2.7 mmol ethyl pentafluoropropionate delivering the titled compound **7h** (521 mg, 61%). ^1^H NMR (400 MHz, CDCl_3_): δ/ppm 8.72 (d, ^4^*J* = 2.3 Hz, 1H), 7.83 (dd, ^3^*J* = 8.9 Hz, ^4^*J* = 1.8 Hz 1H), 6.43 (m, 2H), 5.89 (s, 1H), 3.53 (m, 4H), 3.37 (s, 3H), 1.92 (qui, ^3^*J* = 6.1 Hz, 2H). ^13^C NMR (101 MHz, CDCl_3_): δ/ppm 185.4 (1C), 175.8 (1C), 161.5 (1C), 150.9 (1C), 136.6 (1C), 118.4 (1C), 117.8 (1C), 108.0 (1C), 106.7 (1C), 92.3 (1C), 71.4 (1C), 59.0 (1C), 40.6 (1C), 29.1 (1C). MS (ESI negative) *m*/*z* (%): 353 (100) [M – H]^−^, 707 (13) [2M – H]^−^, 729 (9) [2M – 2H + Na]^−^.

#### 4,4,5,5,5-Pentafluoro-3-hydroxy-1-(6-((2,2,2-trifluoroethyl)amino)pyridin-3-yl)pent-2-en-1-one **7i**

Applying the general procedure with 2,2,2-trifluoroethylamine dissolved in 1-methylpyrrolidin-2-one at 230 °C for 45 min resulted in 1-(6-((2,2,2-trifluoroethyl)amino)pyridin-3-yl)ethan-1-one (1.15 g, 45%). 5.3 mmol of the product were reacted with 5.6 mmol ethyl pentafluoropropionate delivering the titled compound **7i** (1.34 g, 70%). ^1^H NMR (400 MHz, DMSO-*d*_6_): δ/ppm 8.92 (d, ^4^*J* = 2.4 Hz, 1H), 8.39 (t, ^3^*J* = 6.4 Hz, 1H), 8.13 (dd, ^3^*J* = 9.0 Hz, ^4^*J* = 2.4 Hz, 1H), 6.94 (s, 1H), 6.77 (d, ^3^*J* = 9.0 Hz, 1H), 4.33 (m, 2H). ^13^C NMR (101 MHz, DMSO-*d*_6_): δ/ppm 185.5 (1C), 173.3 (1C), 161.3 (1C), 151.1 (1C), 136.5 (1C), 125,1 (1C), 118.0 (1C), 118.0 (1C), 109.1 (1C), 107.6 (1C), 93.1 (1C), 41.1 (1C). MS (ESI negative) *m*/*z* (%): 363 (100) [M – H]^−^, 727 (29) [2M – H]^−^, 749 (7) [2M – 2H + Na]^−^.

#### 4,4,5,5,5-Pentafluoro-3-hydroxy-1-(6-(isobutylamino)pyridin-3-yl)pent-2-en-1-one **7j**

Applying the general procedure with isobutylamine resulted in 1-(6-(isobutylamino)pyridin-3-yl)ethan-1-one (1.80 g, 78%). 4.7 mmol of the product were reacted with 5.3 mmol ethyl pentafluoropropionate delivering the titled compound **7j** (1000 mg, 63%). ^1^H NMR (400 MHz, CDCl_3_): δ/ppm 8.72 (d, ^4^*J* = 2.2 Hz, 1H), 7.95 (dd, ^3^*J* = 9.0 Hz, ^4^*J* = 1.6 Hz, 1H), 6.43 (m, 2H), 5.59 (s, 1H), 3.21 (m, 2H), 1.93 (n, ^3^*J* = 6.7 Hz, 1H), 1.00 (d, ^3^*J* = 6.7 Hz, 6H). ^13^C NMR (101 MHz, CDCl_3_): δ/ppm 185.3 (1C), 175.7 (1C), 161.6 (1C), 150.8 (1C), 136.8 (1C), 118.4 (1C), 118.1 (1C), 108.1 (1C), 106.5 (1C), 92.2 (1C), 49.8 (1C), 28.5 (1C), 20.3 (2C). MS (ESI negative) *m*/*z* (%): 337 (100) [M – H]^−^, 675 (16) [2M – H]^−^.

#### 1-(6-(Cyclopropylamino)pyridin-3-yl)-4,4,5,5,5-pentafluoro-3-hydroxypent-2-en-1-one **7k**

1-(6-Chloropyridin-3-yl)ethan-1-one (9 mmol), cyclopropylamine (45 mmol) and potassium carbonate (54 mmol) in DMF were heated to 100 °C for 4 h. DMF was evaporated, ethyl acetate added, washed with brine, and evaporated to dryness. The residue was purified by flash-chromatography resulting in 1-(6-(cyclopropylamino)pyridin-3-yl)ethan-1-one (650 mg, 41%). Applying the general procedure with 1-(6-(cyclopropylamino)pyridin-3-yl)ethan-1-one (3.1 mmol) and ethyl pentafluoropropionate (3.5 mmol) delivered the titled compound **7k** (553 mg, 55%). ^1^H NMR (400 MHz, DMSO-*d*_6_): δ/ppm 8.88 (d, 1H), 8.16 (m, 2H), 6.82 (m, 2H), 2.72 (s, 1H), 0.78 (m, 2H), 0.52 (m, 2H). ^13^C NMR (101 MHz, DMSO-*d*_6_): δ/ppm 185.4 (1C), 173.2 (1C), 163.0 (1C), 151.8 (1C), 136.5 (1C), 118.0 (1C), 116.5 (1C), 107.7 (1C), 105.6 (1C), 92.5 (1C), 23.8 (1C), 6.6 (2C). MS (ESI negative) *m*/*z* (%): 321 (100) [M – H]^−^, 643 (5) [2M – H]^−^, 665 (16) [2M – 2H + Na]^−^.

#### 1-(6-(Cyclobutylamino)pyridin-3-yl)-4,4,5,5,5-pentafluoro-3-hydroxypent-2-en-1-one **7l**

Applying the general procedure with cyclobutylamine and 1-methylpyrrolidin-2-one as solvent at 200 °C for 30 min resulted in 1-(6-(cyclobutylamino)pyridin-3-yl)ethan-1-one (1.95 g, 80%). 9.2 mmol of the product were reacted with 10.3 mmol ethyl pentafluoropropionate delivering the titled compound **7l** (1743 mg, 57%). ^1^H NMR (400 MHz, DMSO-*d*_6_): δ/ppm 8.86 (d, ^4^*J* = 2.4 Hz, 1H), 8.26 (d, ^3^*J* = 6.9 Hz, 1H), 8.01 (m, 1H), 6.86 (s, 1H), 6.52 (d, ^3^*J* = 9.0 Hz, 1H), 4.51 (s, 1H), 2.30 (m, 2H), 1.95 (m, 2H), 1.71 (m, 2H). ^13^C NMR (101 MHz, DMSO-*d*_6_): δ/ppm 185.4 (1C), 173.1 (1C), 160.7 (1C), 152.2 (1C), 135.5 (1C), 118.1 (1C), 115.9 (1C), 109.0 (1C), 107.6 (1C), 92.4 (1C), 45.8 (1C), 30.2 (2C), 14.8 (1C). MS (ESI negative) *m*/*z* (%): 335 (100) [M – H]^−^, 671 (13) [2M – H]^−^, 693 (7) [2M – 2H + Na]^−^.

#### 1-(6-((Cyclopropylmethyl)amino)pyridin-3-yl)-4,4,5,5,5-pentafluoro-3-hydroxypent-2-en-1-one **7m**

Applying the general procedure with cyclopropylmethylamine resulted in 1-(6-(cyclopropylmethylamino)pyridin-3-yl)ethan-1-one (999 mg, 88%). 2.6 mmol of the product were reacted with 3.0 mmol ethyl pentafluoropropionate delivering the titled compound **7m** (678 mg, 77%). ^1^H NMR (400 MHz, DMSO-*d*_6_): δ/ppm 8.86 (d, ^4^*J* = 2.4 Hz, 1H), 8.12 (t, 1H), 8.0 (d, ^3^*J* = 8.1 Hz, 1H), 6.86 (s, 1H), 6.60 (d, ^3^*J* = 9.1 Hz 1H), 3.27 (m, 2H), 1.05 (m, 1H), 0.47 (m, 2H), 0.24 (m, 2H). ^13^C NMR (101 MHz, DMSO-*d*_6_): δ/ppm 185.4 (1C), 173.1 (1C), 161.6 (1C), 152.2 (1C), 135.4 (1C), 118.0 (1C), 115.6 (1C), 109.2 (1C), 107.7 (1C), 92.3 (1C), 45.2 (1C), 10.6 (1C), 3.4 (2C). MS (ESI negative) *m*/*z* (%): 335 (100) [M – H]^−^, 671 (16) [2M – H]^−^, 693 (10) [2M – 2H + Na]^−^.

#### 4,4,5,5,5-Pentafluoro-3-hydroxy-1-(6-(isopropylamino)pyridin-3-yl)pent-2-en-1-one **7n**

Applying the general procedure with isopropylamine resulted in 1-(6-(isopropylamino)pyridin-3-yl)ethan-1-one (1.17 g, 82%). 2.8 mmol of the product were reacted with 3.2 mmol ethyl pentafluoropropionate delivering the titled compound **7n** (647 mg, 71%). ^1^H NMR (400 MHz, CDCl_3_): δ/ppm 8.73 (d, ^4^*J* = 2.3 Hz, 1H), 7.95 (dd, ^3^*J* = 9.0 Hz, ^4^*J* = 2.3 Hz, 1H), 6.45 (s, 1H), 6.41 (d, ^3^*J* = 9.0 Hz, 1H), 5.40 (s, 1H), 4.03 (m, 1H), 1.28 (d, ^3^*J* = 6.4, 6H). ^13^C NMR (101 MHz, CDCl_3_): δ/ppm 185.3 (1C), 175.7 (1C), 160.6 (1C), 150.8 (1C), 136.8 (1C), 118.6 (1C), 118.0 (1C), 108.1 (1C), 106.7 (1C), 92.2 (1C), 43.8 (1C), 22.8 (2C). MS (ESI negative) *m*/*z* (%): 323 (100) [M – H]^−^, 669 (16) [2M – 2H + Na]^−^.

#### 1-(6-(*tert*-Butylamino)pyridin-3-yl)-4,4,5,5,5-pentafluoro-3-hydroxypent-2-en-1-one **7o**

Applying the general procedure with *tert*-butylamine dissolved in *n*-butanol at 160 °C for 90 min resulted in 1-(6-(*tert*-butylamino)pyridin-3-yl)ethan-1-one (1.47 g, 48%). 5.0 mmol of the product were reacted with 5.6 mmol ethyl pentafluoropropionate delivering the titled compound **7o** (1.09 g, 65%). ^1^H NMR (400 MHz, DMSO-*d*_6_): δ/ppm 8.88 (d, ^4^*J* = 2.5 Hz, 1H), 7.94 (dd, ^3^*J* = 9.1 Hz, ^4^*J* = 2.5 Hz, 1H), 7.68 (s, 1H), 6.88 (s, 1H), 6.62 (d, ^3^*J* = 9.1 Hz, 1H), 1.43 (s, 9H). ^13^C NMR (101 MHz, DMSO-*d*_6_): δ/ppm 185.7 (1C), 173.0 (1C), 161.7 (1C), 151.6 (1C), 134.8 (1C), 118.0 (1C), 115.2 (1C), 110.3 (1C), 107.6 (1C), 92.4 (1C), 51.6 (1C), 28.6 (3C). MS (ESI negative) *m*/*z* (%): 337 (100) [M – H]^−^, 675 (7) [2M – H]^−^, 697 (58) [2M – 2H + Na]^−^.

#### 4,4,5,5,5-Pentafluoro-1-(6-((4-fluorobenzyl)amino)pyridin-3-yl)-3-hydroxypent-2-en-1-one **7p**

Applying the general procedure with 4-fluorobenzylamine at 140 °C resulted in 1-(6-(4-fluorobenzylamino)pyridin-3-yl)ethan-1-one (893 mg, 41%). 2.7 mmol of the product were reacted with 3.0 mmol ethyl pentafluoropropionate delivering the titled compound **7p** (716 mg, 69%). ^1^H NMR (400 MHz, CDCl_3_): δ/ppm 15.60 (br s, 1H), 8.71 (d, ^4^*J* = 2.2 Hz, 1H), 7.95 (dd, ^4^*J* = 2.3 Hz, ^3^*J* = 8.9 Hz, 1H), 7.31 (m, 2H), 7.04 (m, 2H), 6.44 (m, 2H), 5.78 (s, 1H), 4.60 (d, ^4^*J* = 5.8 Hz, 2H). ^13^C NMR (101 MHz, CDCl_3_): δ/ppm 185.2 (1C), 176.1 (1C), 162.5 (1C), 161.2 (1C), 150.7 (1C), 136.8 (1C), 133.5 (1C), 129.3 (2C), 118.7 (1C), 118.2 (1C), 115.9 (2C), 108.0 (1C), 107.0 (1C), 92.4 (1C), 45.5 (1C). MS (ESI negative) *m*/*z* (%): 389 (100) [M – H]^−^, 779 (50) [2M – H]^−^, 801 (6) [2M – 2H + Na]^−^.

#### 4,4,5,5,5-Pentafluoro-3-hydroxy-1-(6-((3,4,5-trimethoxybenzyl)amino)pyridin-3-yl)pent-2-en-1-one **7q**

Applying the general procedure with 3,4,5-trimethoxybenzylamine (3.2 mmol) and 1-(6-bromopyridin-3-yl)ethanone (2.2 mmol) at 100 °C for 10 min resulted in 1-(6-((3,4,5-trimethoxybenzyl)amino)pyridin-3-yl)ethan-1-one (155 mg, 18%). 0.46 mmol of the product were reacted with 0.52 mmol ethyl pentafluoropropionate delivering the titled compound **7q** (88 mg, 41%). ^1^H NMR (400 MHz, DMSO-*d*_6_): δ/ppm 8.90 (d, ^4^*J* = 2.0 Hz, 1H), 8.39 (t, 1H), 8.05 (d, ^3^*J* = 8.7 Hz, 1H), 6.89 (s, 1H), 6.66 (m, 3H), 4.55 (s, 2H), 3.74 (s, 6H), 3.63 (s, 3H). ^13^C NMR (101 MHz, DMSO-*d*_6_): δ/ppm 185.4 (1C), 173.2 (1C), 161.5 (1C), 152.9 (2C), 152.0 (1C), 136.5 (1C), 135.6 (1C), 134.5 (1C), 118.1 (1C), 116.2 (1C), 109.4 (1C), 107.6 (1C), 104.9 (2C), 92.5 (1C), 60.0 (1C), 55.8 (2C), 44.5 (1C). MS (ESI negative) *m*/*z* (%): 461 (100) [M – H]^−^.

#### 4,4,5,5,5-Pentafluoro-3-hydroxy-1-(6-(methyl(propyl)amino)pyridin-3-yl)pent-2-en-1-one **7r**

Applying the general procedure with *n*-methylpropylamine resulted in 1-(6-(methyl(propyl)amino)pyridin-3-yl)ethan-1-one (1073 mg, 93%). 2.6 mmol of the product were reacted with 2.9 mmol ethyl pentafluoropropionate delivering the titled compound **7r** (478 mg, 54%). ^1^H NMR (400 MHz, DMSO-*d*_6_): δ/ppm 8.92 (d, ^4^*J* = 2.4 Hz, 1H), 8.11 (dd, ^3^*J* = 9.2 Hz, ^4^*J* = 2.5 Hz, 1H), 6.91 (s, 1H), 6.77 (d, ^3^*J* = 9.2 Hz, 1H), 3.61 (t, 2H), 3.14 (s, 3H), 1.59 (sxt, ^3^*J* = 7.4 Hz, 2H), 0.87 (t, ^3^*J* = 7.4 Hz, 3H). ^13^C NMR (101 MHz, DMSO-*d*_6_): δ/ppm 185.2 (1C), 173.3 (1C), 160.6 (1C), 151.4 (1C), 136.3 (1C), 118.1 (1C), 115.2 (1C), 107.6 (1C), 105.8 (1C), 92.5 (1C), 51.1 (1C), 36.3 (1C), 20.0 (1C), 11.1 (1C). MS (ESI negative) *m*/*z* (%): 337 (100) [M – H]^−^, 697 (15) [2M – 2H + Na]^−^.

#### 1-(6-(Diethylamino)pyridin-3-yl)-4,4,5,5,5-pentafluoro-3-hydroxypent-2-en-1-one **7s**

1-(6-Chloropyridin-3-yl)ethan-1-one (6.75 mmol), diethylamine (67 mmol) and potassium carbonate (40 mmol) in DMF were heated to 110 °C for 12 h. DMF was evaporated, and the residue dissolved in DCM, washed with brine, dried over Na_2_SO_4_, filtered, and evaporated to dryness resulting in 1-(6-(diethylamino)pyridin-3-yl)ethan-1-one (1.29 g, 99%). Applying the general procedure with 1-(6-(diethylamino)pyridin-3-yl)ethan-1-one (6.76 mmol) and ethyl pentafluoropropionate (7.61 mmol) delivered the titled compound **7s** (1.71 g, 75%). ^1^H NMR (400 MHz, DMSO-*d*_6_): δ/ppm 8.92 (d, ^4^*J* = 2.4 Hz, 1H), 8.10 (dd, ^3^*J* = 9.3 Hz, ^4^*J* = 2.5 Hz, 1H), 6.90 (s, 1H), 6.75 (d, 1H, ^3^*J* = 9.3 Hz), 3.61 (q, ^3^*J* = 6.6 Hz, 4H), 1.15 (t, ^3^*J* = 7.0 Hz, 6H). ^13^C NMR (101 MHz, DMSO-*d*_6_): δ/ppm 185.2 (1C), 173.3 (1C), 159.6 (1C), 151.7 (1C), 136.4 (1C), 118.1 (1C), 115.1 (1C), 107.6 (1C), 105.7 (1C), 92.4 (1C), 42.6 (2C), 12.7 (2C). MS (ESI negative) *m*/*z* (%): 337 (100) [M – H]^−^, 697 (17) [2M – 2H + Na]^−^.

#### 4,4,5,5,5-Pentafluoro-3-hydroxy-1-[6-(piperazin-1-yl)pyridin-3-yl]pent-2-en-1-one **7t**

Applying the general procedure with piperazine and extraction at pH 9 of the aqueous phase resulted in 1-(6-(piperazin-1-yl)pyridin-3-yl)ethan-1-one (345 mg, 47%). 0.84 mmol of the product were reacted with 0.92 mmol ethyl pentafluoropropionate delivering the titled compound **7t** (110 mg, 37%). ^1^H NMR (400 MHz, DMSO-*d*_6_): δ/ppm 9.58 (s, 1H), 8.57 (d, ^4^*J* = 2.3 Hz, 1H), 7.93 (dd, ^3^*J* = 8.9 Hz, ^4^*J* = 2.3 Hz, 1H), 6.86 (d, ^3^*J* = 8.9 Hz, 1H), 5.85 (s, 1H), 3.78 (m, 4H), 3.20 (m, 4H). ^13^C NMR (101 MHz, DMSO-*d*_6_): δ/ppm 182.8 (1C), 168.7 (1C), 158.8 (1C), 147.3 (1C), 136.5 (1C), 126.7 (1C), 119.1 (1C), 108.9 (1C), 106.1 (1C), 87.8 (1C), 42.6 (2C), 41.6 (2C). MS (ESI negative) *m*/*z* (%): 350 (100) [M – H]^−^.

#### *N*-[5-(4,4,5,5,5-Pentafluoro-3-hydroxypent-2-enoyl)pyridin-2-yl]acetamide **8a**

Compound **7b** (1.31 mmol) was suspended in pyridine, treated with acetic anhydride (4.32 mmol), and stirred at 60 °C for 9 h. The solvent was evaporated, the residue was dissolved in ethyl acetate, and washed with brine. After evaporation of the organic phase and flash-chromatography the titled compound **8a** (179 mg, 42%) was delivered. ^1^H NMR (400 MHz, DMSO-*d*_6_): δ/ppm 11.06 (s, 1H), 9.09 (d, ^4^*J* = 2.4 Hz, 1H), 8.46 (dd, ^4^*J* = 2.4 Hz, ^3^*J* = 8.9 Hz, 1H), 8.23 (d, ^3^*J* = 8.9, 1H), 7.08 (s, 1H), 2.15 (s, 3H). ^13^C NMR (101 MHz, DMSO-*d*_6_): δ/ppm 183.9 (1C), 174.6 (1C), 170.1 (1C), 156.1 (1C), 149.3 (1C), 138.1 (1C), 123.6 (1C), 117.9 (1C), 112.5 (1C), 107.5 (1C), 94.2 (1C), 24.6 (1C). MS (ESI negative) *m*/*z* (%): *m*/*z* (%): 323 (100) [M – H]^−^, 669 (19) [2M – 2H + Na]^−^

#### *N*-(5-(4,4,5,5,5-Pentafluoro-3-hydroxypent-2-enoyl)pyridin-2-yl)propionamide **8b**

Compound **7b** (3.53 mmol) was suspended in pyridine, treated with propionic anhydride (11.6 mmol), and stirred at 60 °C for 13 h. The solvent was evaporated, the residue was dissolved in ethyl acetate, and washed with brine. After evaporation of the organic phase and flash-chromatography the titled compound **8b** (254 mg, 21%) was delivered. ^1^H NMR (400 MHz, DMSO-*d*_6_): δ/ppm 11.01 (s, 1H), 9.08 (d, ^4^*J* = 2.2 Hz, 1H), 8.45 (dd, ^3^*J* = 8.9 Hz, ^4^*J* = 2.4 Hz, 1H), 8.25 (d, ^3^*J* = 8.9 Hz, 1H), 7.08 (s, 1H), 2.46 (q, ^3^*J* = 7.5 Hz, 2H), 1.07 (t, ^3^*J* = 7.5 Hz, 3H). ^13^C NMR (101 MHz, DMSO-*d*_6_): δ/ppm 184.0 (1C), 174.6 (1C), 173.7 (1C), 156.1 (1C), 149.4 (1C), 138.2 (1C), 123.5 (1C), 118.0 (1C), 112.5 (1C), 107.5 (1C), 94.2 (1C), 29.5 (1C), 9.1 (1C). MS (ESI negative) *m*/*z* (%): 337 (100) [M – H]^−^, 697 (25) [2M – 2H + Na]^−^.

#### *N*-(5-(4,4,5,5,5-Pentafluoro-3-hydroxypent-2-enoyl)pyridin-2-yl)benzamide **8c**

Compound **7b** (1.77 mmol) was suspended in pyridine, treated with propionic anhydride (3.89 mmol), and stirred at 80 °C for 9 h. The solvent was evaporated, the residue was dissolved in ethyl acetate, and washed with brine. After evaporation of the organic phase and flash-chromatography the titled compound **8c** (252 mg, 37%) was delivered. ^1^H NMR (400 MHz, DMSO-*d*_6_): δ/ppm 11.36 (s, 1H), 9.16 (d, ^4^*J* = 2.2 Hz, 1H), 8.54 (dd, ^3^*J* = 8.9 Hz, ^4^*J* = 2.3 Hz, 1H), 8.39 (d, ^3^*J* = 8.9 Hz, 1H), 8.05 (m, 2H), 7.63 (m, 1H), 7.53 (m, 2H), 7.12 (s, 1H). ^13^C NMR (101 MHz, DMSO-*d*_6_): δ/ppm 183.7 (1C), 174.8 (1C), 166.6 (1C), 156.4 (1C), 149.1 (1C), 138.1 (1C), 133.5 (1C), 132.4 (1C), 128.4 (2C), 128.3 (2C), 124.2 (1C), 118.0 (1C), 113), 107.5 (1C), 94.3 (1C). MS (ESI negative) *m*/*z* (%): 385 (100) [M – H]^−^, 793 (10) [2M – 2H + Na]^−^.

## Supporting Information Available

*K*_*i*_ and IC_50_ curves of all compounds assayed in this study, purity of **7e** and **8a** by HPLC (PDF)
Compound SMILES list (CSV)

## Abbreviation

PfFNT: plasmodium falciparum formate-nitrite transporter

## References

[ref1] Wicht K. J.; Mok S.; Fidock D. A. Molecular mechanisms of drug resistance in Plasmodium falciparum malaria. Annu. Rev. Microbiol. 2020, 74, 431–454. 10.1146/annurev-micro-020518-115546. 32905757 PMC8130186

[ref2] Pan H. Z.; Lin F. B.; Zhang Z. A. Effect of sodium artesunate on malaria infected human erythrocytes. Proc. Chin. Acad. Med. Sci. Peking Union Med. Coll. 1989, 4, 181–185. 2698476

[ref3] Spillman N. J.; Allen R. J.; McNamara C. W.; Yeung B. K.; Winzeler E. A.; Diagana T. T.; Kirk K. Na^+^ regulation in the malaria parasite Plasmodium falciparum involves the cation ATPase PfATP4 and is a target of the spiroindolone antimalarials. Cell Host Microbe 2013, 13, 227–237. 10.1016/j.chom.2012.12.006. 23414762 PMC3574224

[ref4] Golldack A.; Henke B.; Bergmann B.; Wiechert M.; Erler H.; Blancke Soares A.; Spielmann T.; Beitz E. Substrate-analogous inhibitors exert antimalarial action by targeting the Plasmodium lactate transporter PfFNT at nanomolar scale. PLoS Pathog. 2017, 13, e1006172 10.1371/journal.ppat.1006172. 28178358 PMC5298233

[ref5] Hapuarachchi S. V.; Cobbold S. A.; Shafik S. H.; Dennis A. S.; McConville M. J.; Martin R. E.; Kirk K.; Lehane A. M. The malaria parasite’s lactate transporter PfFNT is the target of antiplasmodial compounds identified in whole cell phenotypic screens. PLoS Pathog. 2017, 13, e1006180 10.1371/journal.ppat.1006180. 28178359 PMC5298231

[ref6] Walloch P.; Henke B.; Häuer S.; Bergmann B.; Spielmann T.; Beitz E. Introduction of scaffold nitrogen atoms renders inhibitors of the malarial l-lactate transporter, PfFNT, effective against the Gly107Ser resistance mutation. J. Med. Chem. 2020, 63, 9731–9741. 10.1021/acs.jmedchem.0c00852. 32816478

[ref7] Walloch P.; Hansen C.; Priegann T.; Schade D.; Beitz E. Pentafluoro-3-hydroxy-pent-2-en-1-ones potently inhibit FNT-type lactate transporters from all five human-pathogenic Plasmodium species. ChemMedChem 2021, 16, 1283–1289. 10.1002/cmdc.202000952. 33336890 PMC8247949

[ref8] Wu B.; Rambow J.; Bock S.; Holm-Bertelsen J.; Wiechert M.; Soares A. B.; Spielmann T.; Beitz E. Identity of a Plasmodium lactate/H^+^ symporter structurally unrelated to human transporters. Nat. Commun. 2015, 6, 6284 10.1038/ncomms7284. 25669138

[ref9] Marchetti R. V.; Lehane A. M.; Shafik S. H.; Winterberg M.; Martin R. E.; Kirk K. A Lactate and formate transporter in the intraerythrocytic malaria parasite, Plasmodium falciparum. Nat. Commun. 2015, 6, 6721 10.1038/ncomms7721. 25823844

[ref10] Davies H.; Bergmann B.; Walloch P.; Nerlich C.; Hansen C.; Wittlin S.; Spielmann T.; Treeck M.; Beitz E. The Plasmodium lactate/H^+^ transporter PfFNT is essential and druggable in vivo. Antimicrob. Agents Chemother. 2023, 67, e0035623 10.1128/aac.00356-23. 37428074 PMC10433847

[ref11] Peng X.; Wang N.; Zhu A.; Xu H.; Li J.; Zhou Y.; Wang C.; Xiao Q.; Guo L.; Liu F.; Jia Z. J.; Duan H.; Hu J.; Yuan W.; Geng J.; Yan C.; Jiang X.; Deng D. Structural characterization of the Plasmodium falciparum lactate transporter PfFNT alone and in complex with antimalarial compound MMV007839 reveals its inhibition mechanism. PLoS Biol. 2021, 19, e3001386 10.1371/journal.pbio.3001386. 34499638 PMC8428694

[ref12] Van Voorhis W. C.; Adams J. H.; Adelfio R.; Ahyong V.; Akabas M. H.; Alano P.; Alday A.; Alemán Resto Y.; Alsibaee A.; Alzualde A.; Andrews K. T.; Avery S. V.; Avery V. M.; Ayong L.; Baker M.; Baker S.; Ben Mamoun C.; Bhatia S.; Bickle Q.; Bounaadja L.; Bowling T.; Bosch J.; Boucher L. E.; Boyom F. F.; Brea J.; Brennan M.; Burton A.; Caffrey C. R.; Camarda G.; Carrasquilla M.; Carter D.; Belen Cassera M.; Chih-Chien Cheng K.; Chindaudomsate W.; Chubb A.; Colon B. L.; Colón-López D. D.; Corbett Y.; Crowther G. J.; Cowan N.; D’Alessandro S.; Le Dang N.; Delves M.; DeRisi J. L.; Du A. Y.; Duffy S.; Abd El-Salam El-Sayed S.; Ferdig M. T.; Fernández Robledo J. A.; Fidock D. A.; Florent I.; Fokou P. V. T.; Galstian A.; Gamo F. J.; Gokool S.; Gold B.; Golub T.; Goldgof G. M.; Guha R.; Guiguemde W. A.; Gural N.; Guy R. K.; Hansen M. A. E.; Hanson K. K.; Hemphill A.; Hooft van Huijsduijnen R.; Horii T.; Horrocks P.; Hughes T. B.; Huston C.; Igarashi I.; Ingram-Sieber K.; Itoe M. A.; Jadhav A.; Naranuntarat Jensen A.; Jensen L. T.; Jiang R. H. Y.; Kaiser A.; Keiser J.; Ketas T.; Kicka S.; Kim S.; Kirk K.; Kumar V. P.; Kyle D. E.; Lafuente M. J.; Landfear S.; Lee N.; Lee S.; Lehane A. M.; Li F.; Little D.; Liu L.; Llinás M.; Loza M. I.; Lubar A.; Lucantoni L.; Lucet I.; Maes L.; Mancama D.; Mansour N. R.; March S.; McGowan S.; Medina Vera I.; Meister S.; Mercer L.; Mestres J.; Mfopa A. N.; Misra R. N.; Moon S.; Moore J. P.; Morais Rodrigues da Costa F.; Müller J.; Muriana A.; Nakazawa Hewitt S.; Nare B.; Nathan C.; Narraidoo N.; Nawaratna S.; Ojo K. K.; Ortiz D.; Panic G.; Papadatos G.; Parapini S.; Patra K.; Pham N.; Prats S.; Plouffe D. M.; Poulsen S.-A.; Pradhan A.; Quevedo C.; Quinn R. J.; Rice C. A.; Abdo Rizk M.; Ruecker A.; St Onge R.; Salgado Ferreira R.; Samra J.; Robinett N. G.; Schlecht U.; Schmitt M.; Silva Villela F.; Silvestrini F.; Sinden R.; Smith D. A.; Soldati T.; Spitzmüller A.; Stamm S. M.; Sullivan D. J.; Sullivan W.; Suresh S.; Suzuki B. M.; Suzuki Y.; Swamidass S. J.; Taramelli D.; Tchokouaha L. R. Y.; Theron A.; Thomas D.; Tonissen K. F.; Townson S.; Tripathi A. K.; Trofimov V.; Udenze K. O.; Ullah I.; Vallieres C.; Vigil E.; Vinetz J. M.; Voong Vinh P.; Vu H.; Watanabe N.; Weatherby K.; White P. M.; Wilks A. F.; Winzeler E. A.; Wojcik E.; Wree M.; Wu W.; Yokoyama N.; Zollo P. H. A.; Abla N.; Blasco B.; Burrows J.; Laleu B.; Leroy D.; Spangenberg T.; Wells T.; Willis P. A. Open source drug discovery with the malaria box compound collection for neglected diseases and beyond. PLoS Pathog. 2016, 12, e1005763 10.1371/journal.ppat.1005763. 27467575 PMC4965013

[ref13] Helmstetter F.; Arnold P.; Höger B.; Petersen L. M.; Beitz E. Formate-nitrite transporters carrying nonprotonatable amide amino acids instead of a central histidine maintain pH-dependent transport. J. Biol. Chem. 2019, 294, 623–631. 10.1074/jbc.RA118.006340. 30455351 PMC6333897

[ref14] aSchmidt J. D. R.; Beitz E. Mutational widening of constrictions in a formate-nitrite/H^+^ transporter enables aquaporin-like water permeability and proton conductance. J. Biol. Chem. 2022, 298, 101513 10.1016/j.jbc.2021.101513. 34929166 PMC8749060

[ref15] Schiebel J.; Gaspari R.; Wulsdorf T.; Ngo K.; Sohn C.; Schrader T. E.; Cavalli A.; Ostermann A.; Heine A.; Klebe G. Intriguing role of water in protein-ligand binding studied by neutron crystallography on trypsin complexes. Nat. Commun. 2018, 9, 3559 10.1038/s41467-018-05769-2. 30177695 PMC6120877

[ref16] Angulo-Barturen I.; Jiménez-Díaz M. B.; Mulet T.; Rullas J.; Herreros E.; Ferrer S.; Jiménez E.; Mendoza A.; Regadera J.; Rosenthal P. J.; Bathurst I.; Pompliano D. L.; Gómez de las Heras F.; Gargallo-Viola D. A murine model of falciparuM–malaria by in vivo selection of competent strains in non-myelodepleted mice engrafted with human erythrocytes. PLoS One 2008, 3, e2252 10.1371/journal.pone.0002252. 18493601 PMC2375113

[ref17] Jiménez-Díaz M. B.; Mulet T.; Viera S.; Gómez V.; Garuti H.; Ibáñez J.; Alvarez-Doval A.; Shultz L. D.; Martínez A.; Gargallo-Viola D.; Angulo-Barturen I. Improved murine model of malaria using Plasmodium falciparum competent strains and non-myelodepleted NOD-scid IL2Rgammanull mice engrafted with human erythrocytes. Antimicrob. Agents Chemother. 2009, 53, 4533 10.1128/AAC.00519-09. 19596869 PMC2764183

[ref18] Fidock D.; Rosenthal P. J.; Croft S. L.; Brun R.; Nwaka S. Antimalarial drug discovery: efficacy models for compound screening. Nat. Rev. Drug Discovery 2004, 3, 509–520. 10.1038/nrd1416. 15173840

[ref19] Charman S. A.; Arbe-Barnes S.; Bathurst I. C.; Brun R.; Campbell M.; Charman W. N.; Chiu F. C.; Chollet J.; Craft J. C.; Creek D. J.; Dong Y.; Matile H.; Maurer M.; Morizzi J.; Nguyen T.; Papastogiannidis P.; Scheurer C.; Shackleford D. M.; Sriraghavan K.; Stingelin L.; Tang Y.; Urwyler H.; Wang X.; White K. L.; Wittlin S.; Zhou L.; Vennerstrom J. L. Synthetic ozonide drug candidate OZ439 offers new hope for a single-dose cure of uncomplicated malaria. Proc. Natl. Acad. Sci. U.S.A. 2011, 108, 4400–4405. 10.1073/pnas.1015762108. 21300861 PMC3060245

[ref20] Jakobowska I.; Becker F.; Minguzzi S.; Hansen K.; Henke B.; Epalle N. H.; Beitz E.; Hannus S. Fluorescence cross-correlation spectroscopy yields true affinity and binding kinetics of Plasmodium lactate transport inhibitors. Pharmaceuticals 2021, 14, 757 10.3390/ph14080757. 34451854 PMC8399565

[ref21] Duff M. R.; Howell E. E. Thermodynamics and solvent linkage of macromolecule-ligand interactions. Methods 2015, 76, 51–60. 10.1016/j.ymeth.2014.11.009. 25462561 PMC4380819

[ref22] White N. J. Clinical pharmacokinetics of antimalarial drugs. Clin. Pharmacokinet. 1985, 10, 187–215. 10.2165/00003088-198510030-00001. 3893840

[ref23] Mesén-Ramírez P.; Bergmann B.; Elhabiri M.; Zhu L.; von Thien H.; Castro-Peña C.; Gilberger T. W.; Davioud-Charvet E.; Bozdech Z.; Bachmann A.; Spielmann T. The parasitophorous vacuole nutrient channel is critical for drug access in malaria parasites and modulates the artemisinin resistance fitness cost. Cell Host Microbe 2021, 29, 1774–1787.e9. 10.1016/j.chom.2021.11.002. 34863371

[ref24] Henshall I. G.; Spielmann T. Critical interdependencies between Plasmodium nutrient flux and drugs. Trends Parasitol. 2023, 39, 936–944. 10.1016/j.pt.2023.08.008. 37716852 PMC10580322

[ref25] van de Waterbeemd H.; Smith D. A.; Jones B. C. Lipophilicity in PK design: methyl, ethyl, futile. J. Comput. Aided Mol. Des. 2001, 15, 273–286. 10.1023/A:1008192010023. 11289080

[ref26] Hall A.; Chatzopoulou M.; Frost J. Bioisoteres for carboxylic acids: from ionized isosteres to novel unionized replacements. Bioorg. Med. Chem. 2024, 104, 117653 10.1016/j.bmc.2024.117653. 38579492

[ref27] Segall M. D. Multi-parameter optimization: identifying high quality compounds with a balance of properties. Curr. Pharm. Des. 2012, 18, 1292 10.2174/138161212799436430. 22316157

[ref28] Eh-Haj B. M. Metabolic N-dealkylation and N-oxidation as elucidators of the role of alkylamino moieties in drugs acting at various receptors. Molecules 2021, 26, 1917 10.3390/molecules26071917. 33805491 PMC8036657

[ref29] Hartman J. H.; Knott K.; Miller G. P. CYP2E1 hydroxylation of aniline involves negative cooperativity. Biochem. Pharmacol. 2014, 87, 523–533. 10.1016/j.bcp.2013.12.003. 24345333

[ref30] Antoine T.; Ott D.; Ebell K.; Hansen K.; Henry L.; Becker F.; Hannus S. Homogeneous time-resolved G protein-coupled receptor-ligand binding assay based on fluorescence cross-correlation spectroscopy. Anal. Biochem. 2016, 502, 24–35. 10.1016/j.ab.2016.02.017. 26954998

[ref31] Cheng Y.-C.; Prusoff W. H. Relationship between the inhibition constant (K1) and the concentration of inhibitor which causes 50% inhibition (I50) of an enzymatic reaction. Biochem. Pharmacol. 1973, 22, 3099–3108. 10.1016/0006-2952(73)90196-2. 4202581

[ref32] Soares-Silva I.; Paiva S.; Diallinas G.; Casal M. The conserved sequence NXX[S/T]HX[S/T]QDXXXT of the lactate/pyruvate:H^+^ symporter subfamily defines the function of the substrate translocation pathway. Mol. Membr. Biol. 2007, 24, 464–474. 10.1080/09687680701342669. 17710650

[ref33] Trager W.; Jensen J. B. Human malaria parasites in continuous culture. Science 1976, 193, 673–675. 10.1126/science.781840. 781840

[ref34] Birnbaum J.; Flemming S.; Reichard N.; Soares A. B.; Mesén-Ramírez P.; Jonscher E.; Bergmann B.; Spielmann T. A genetic system to study Plasmodium falciparum protein function. Nat. Methods 2017, 14, 450–456. 10.1038/nmeth.4223. 28288121

[ref35] Malleret B.; Claser C.; Ong A. S.; Suwanarusk R.; Sriprawat K.; Howland S. W.; Russell B.; Nosten F.; Rénia L. A rapid and robust tri-color flow cytometry assay for monitoring malaria parasite development. Sci. Rep. 2011, 1, 118 10.1038/srep00118. 22355635 PMC3216599

[ref36] Dziwornu G. A.; Seanego D.; Fienberg S.; Clements M.; Ferreira J.; Sypu V. S.; Samanta S.; Bhana A. D.; Korkor C. M.; Garnie L. F.; Teixeira N.; Wicht K. J.; Taylor D.; Olckers R.; Njoroge M.; Gibhard L.; Salomane N.; Wittlin S.; Mahato R.; Chakraborty A.; Sevilleno N.; Coyle R.; Lee M. C. S.; Godoy L. C.; Pasaje C. F.; Niles J. C.; Reader J.; van der Watt M.; Birkholtz L. M.; Bolscher J. M.; de Bruijni M. H. C.; Coulson L. B.; Basarab G. S.; Ghorpade S. R.; Chibale K. 2,8-Disubstituted-1,5-naphthyridines as dual inhibitors of Plasmodium falciparum phosphatidylinositol-4-kinase and hemozoin formation with in vivo efficacy. J. Med. Chem. 2024, 67, 11401–11420. 10.1021/acs.jmedchem.4c01154. 38918002 PMC11247499

[ref37] Obach R. S. Prediction of human clearance of twenty-nine drugs from hepatic microsomal intrinsic clearance data: an examination of in vitro half-life approach and nonspecific binding to microsomes. Drug Metab. Dispos. 1999, 27, 1350. 10534321

[ref38] Friesner R. A.; Banks J. L.; Murphy R. B.; Halgren T. A.; Klicic J. J.; Mainz D. T.; Repasky M. P.; Knoll E. H.; Shelley M.; Perry J. K.; Shaw D. E.; Francis P.; Shenkin P. S. Glide: a new approach for rapid, accurate docking and scoring. 1. Method and assessment of docking accuracy. J. Med. Chem. 2004, 47, 1739–1749. 10.1021/jm0306430. 15027865

[ref39] Sherman W.; Day T.; Jacobson M. P.; Friesner R. A.; Farid R. Novel procedure for modeling ligand/receptor induced fit effects. J. Med. Chem. 2006, 49, 534–553. 10.1021/jm050540c. 16420040

[ref40] Pettersen E. F.; Goddard T. D.; Huang C. C.; Couch G. S.; Greenblatt D. M.; Meng E. C.; Ferrin T. E. UCSF Chimera–a visualization system for exploratory research and analysis. J. Comput. Chem. 2004, 25, 1605–1612. 10.1002/jcc.20084. 15264254

[ref41] Boehringer M.; Hunziker D.; Kuehne H.; Loeffler B. M.; Subaru R.; Wessel H. P. N-Substituted pyrrolidin derivatives as dipeptidyl peptidase IV inhibitors. WO 2003037327 A1, 2003.

