# Supplementary material for: Addressing the Intracellular Vestibule of the Plasmodial Lactate Transporter PfFNT by p-Substituted Inhibitors Amplifies In Vitro Activity
Source: J Med Chem. 2024 Oct 3;67(20):18368–83. doi: 10.1021/acs.jmedchem.4c01674 (PMC11513924; doi:10.1021/acs.jmedchem.4c01674)
Supplement: Supplementary file 1 — jm4c01674_si_001.pdf [file jm4c01674_si_001.pdf]

Supporting Information

Addressing the Intracellular Vestibule of the Plasmodial Lactate Transporter PfFNT by *p*-Substituted Inhibitors Amplifies *In Vitro* Activity

Cornelius Nerlich[1], Finn Tiedjens[1], Robin Hertel[1], Björn Henke[1], Susan Häuer[1], Lea S. Panitzsch[2], Kerrin Hansen[3], Ole Franck[1], Antonio Mete[4], Didier Leroy[5], Dennis Schade[1], Christian Peifer[1], Stefan Hannus[3], Frank Becker[3], Sergio Wittlin[6,7], Tobias Spielmann[2], Eric Beitz*[1]

[1]Department of Pharmaceutical and Medicinal Chemistry, Christian-Albrechts-University of Kiel, Gutenbergstr. 76, 24118 Kiel, Germany

[2]Bernhard-Nocht-Institute for Tropical Medicine, Bernhard-Nocht-Str. 74, 20359 Hamburg, Germany

[3]Intana Bioscience GmbH, Lochhamer Str. 29a, 82152 Planegg, Germany

[4]Medsyndesign Ltd, ATIC, 5 Oakwood Drive, Loughborough, LE11 3QF, UK

[5]Medicines for Malaria Venture (MMV), R&D Department/Drug Discovery, ICC, 20 Route de Pré Bois, 1215 Geneva 15, Switzerland

[6]Swiss Tropical and Public Health Institute, Kreuzstr. 2, 4123 Allschwil, Switzerland

[7]University of Basel, 4003 Basel, Switzerland

* Correspondence e-mail: ebeitz@pharmazie.uni-kiel.de

Figure S1 Ligand Affinity to PfFNT

Figure S2 Inhibition of PfFNT ^{14}C-Lactate Transport in Yeast

Figure S3 Purity of **7e** and **8a** by HPLC

A

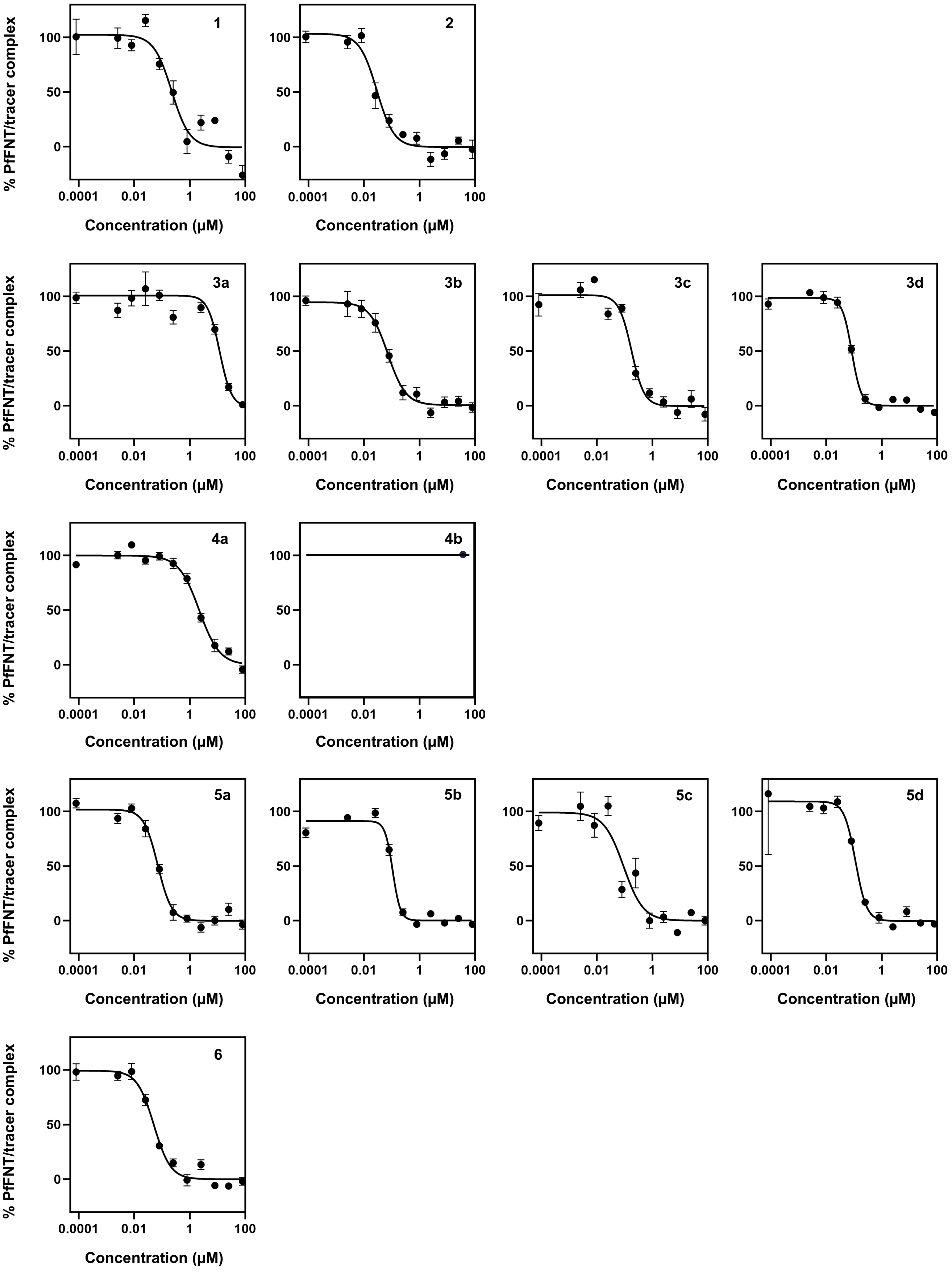
1
2
3a
3b
3c
3d
4a
4b
5a
5b
5c
5d
6
% PfFNT/tracer complex
Concentration (µM)
0.0001
0.01
1
100
0
50
100

B

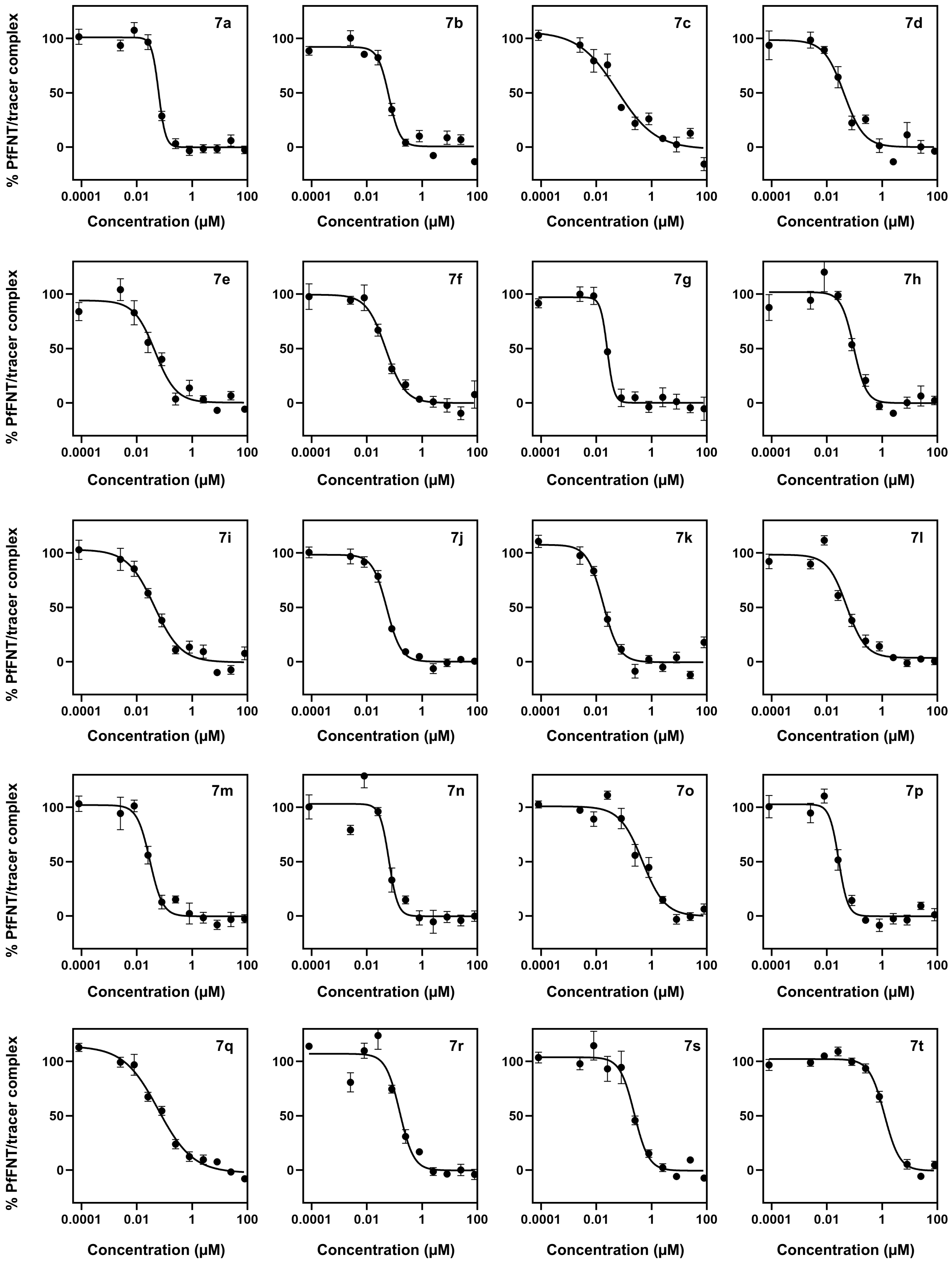
7a
7b
7c
7d
7e
7f
7g
7h
7i
7j
7k
7l
7m
7n
7o
7p
7q
7r
7s
7t
% PfFNT/tracer complex
Concentration (μM)
0.0001
0.01
1
100
0
50
100

C

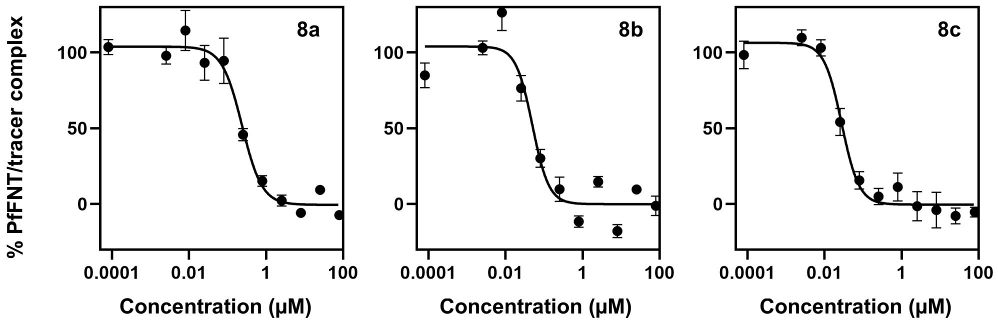

Figure S1. Ligand Affinity to PfFNT. Compounds **1-6**. (**A**), compounds **7a-7t** (**B**), compounds **8a-8c** (**C**). See Methods for K_i calculation.

A

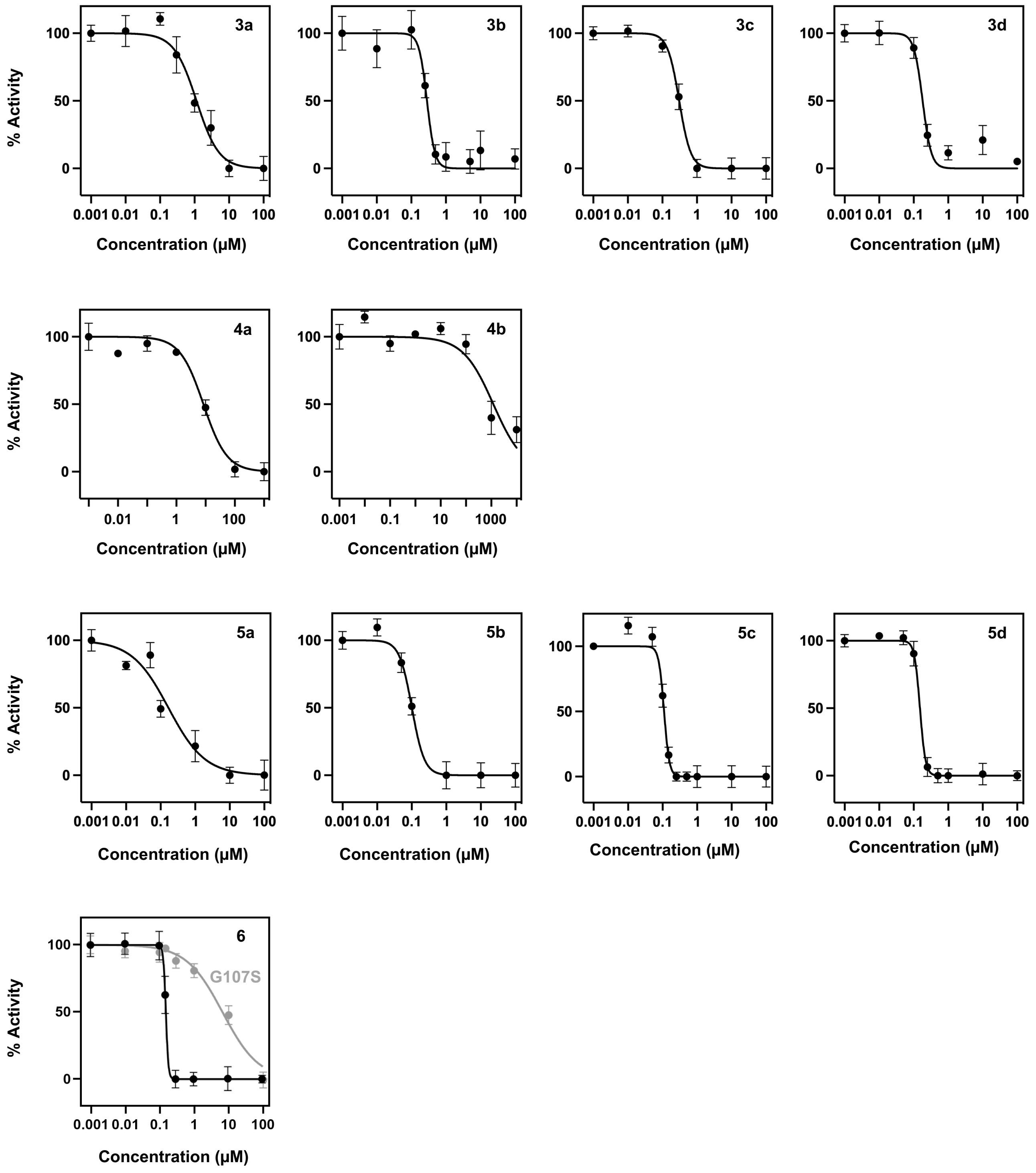
3a
3b
3c
3d
4a
4b
5a
5b
5c
5d
6
G107S
% Activity
100
50
0
0.001 0.01 0.1 1 10 100
0.01 1 100
0.001 0.1 10 1000
Concentration (μM)

B

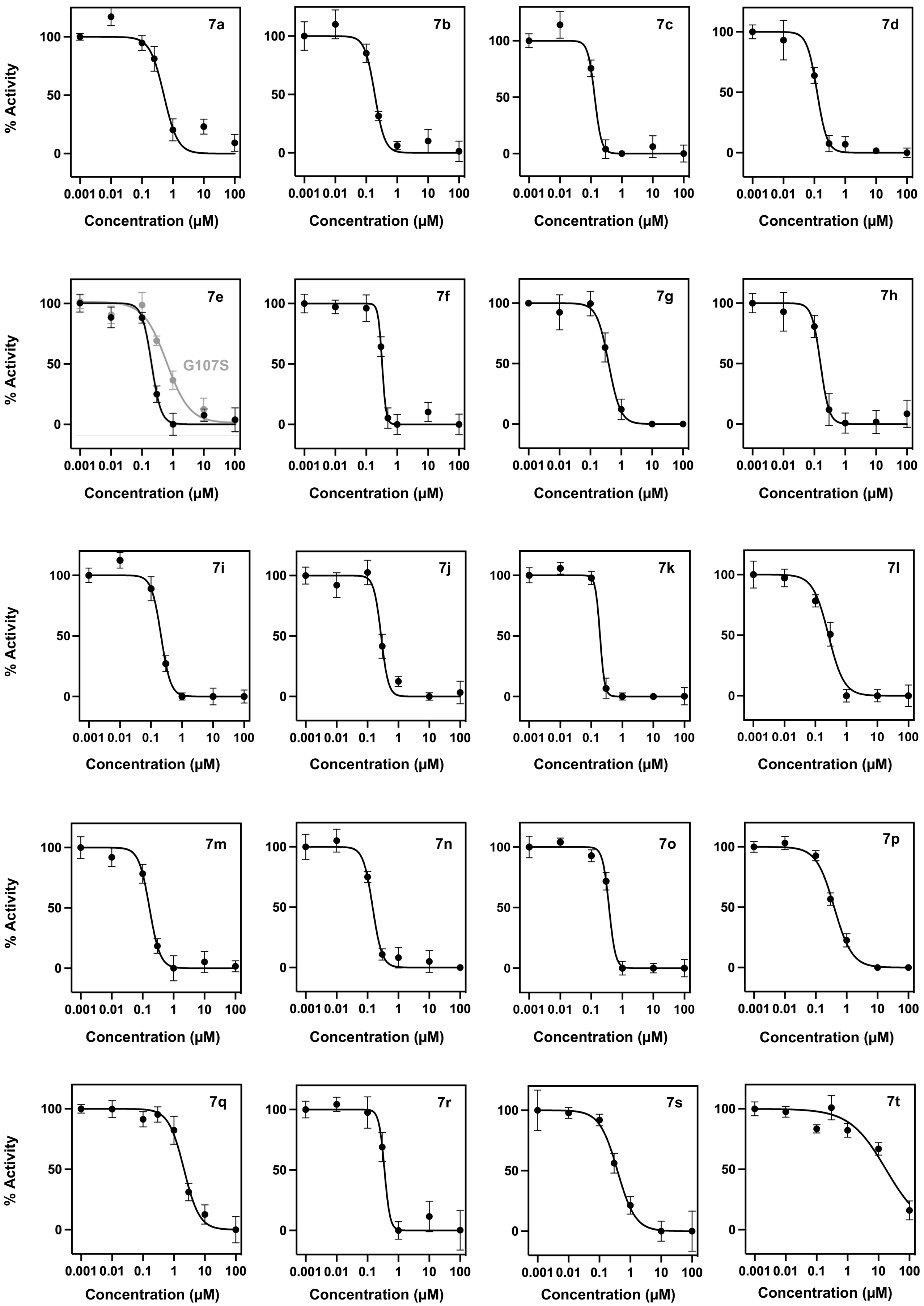
7a
7b
7c
7d
7e
G107S
7f
7g
7h
7i
7j
7k
7l
7m
7n
7o
7p
7q
7r
7s
7t
% Activity
100
50
0
0.001 0.01 0.1 1 10 100
Concentration (μM)

C

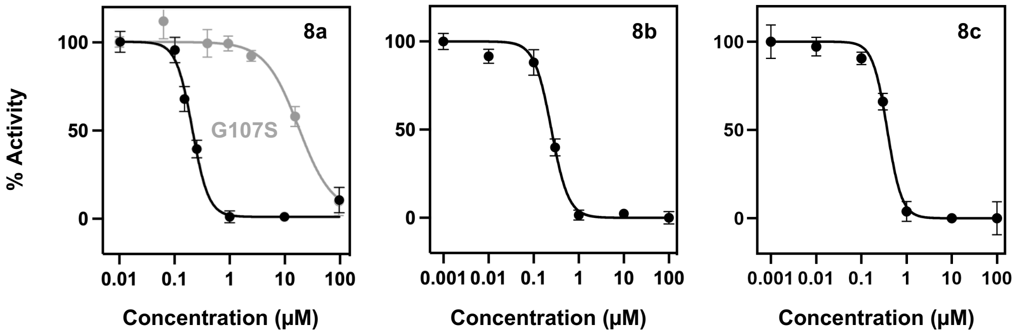

Figure S2. Inhibition of PfFNT ^{14}C-Lactate Transport in Yeast. Compounds **3a-6** (**A**), **c**ompounds **7a-7t** (**B**), **c**ompounds **8a-8c** (**C**). For compounds **6**, **7e**, and **8a**, activity on the PfFNT G107S resistance mutant is shown in addition (grey).

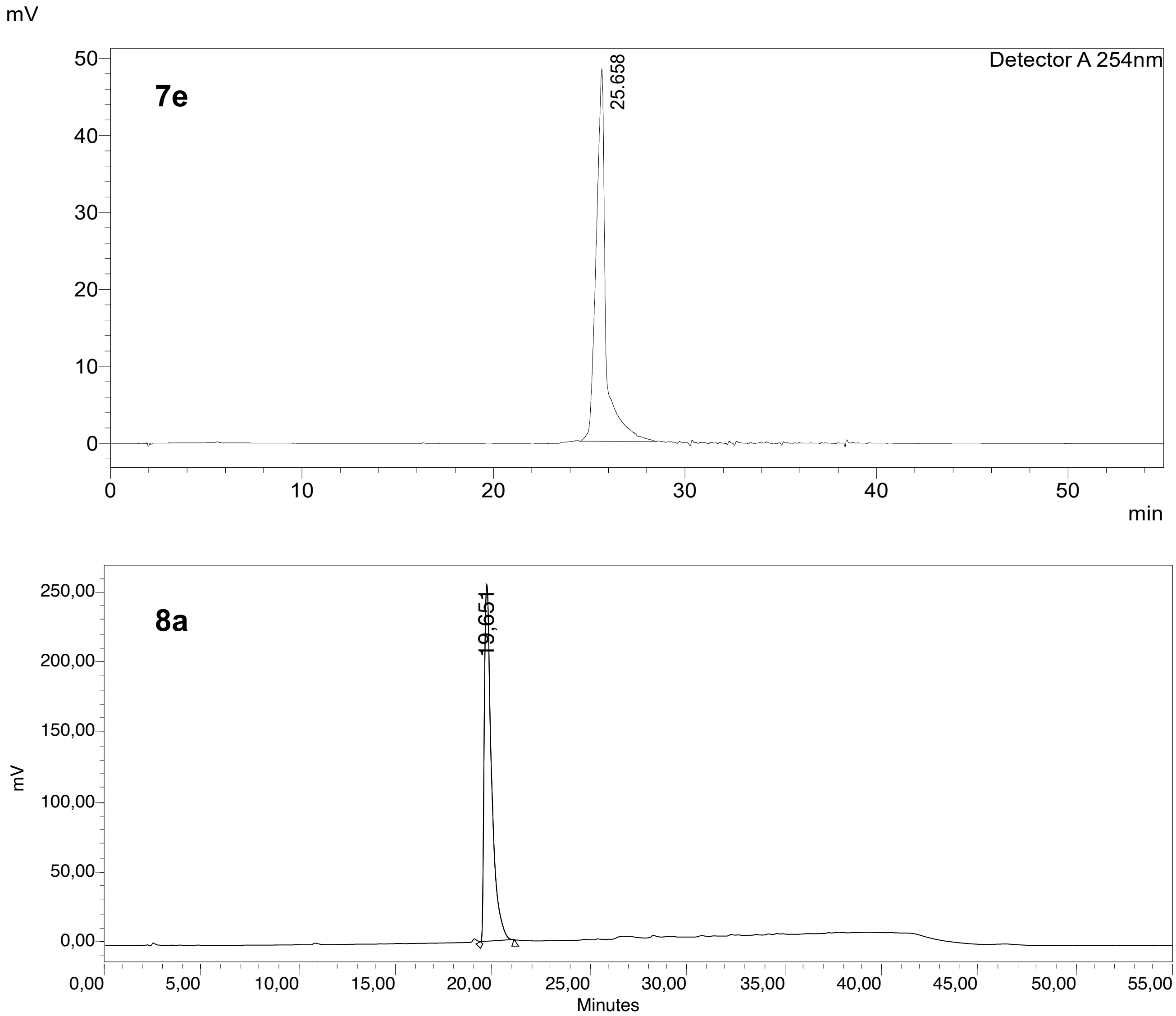

Figure S3. Purity of 7e and 8a by HPLC.
